# Cluster effect for SNP–SNP interaction pairs for predicting complex traits

**DOI:** 10.1038/s41598-024-66311-7

**Published:** 2024-08-12

**Authors:** Hui-Yi Lin, Harun Mazumder, Indrani Sarkar, Po-Yu Huang, Rosalind A. Eeles, Zsofia Kote-Jarai, Kenneth R. Muir, UKGPCS collaborators, Johanna Schleutker, Nora Pashayan, Jyotsna Batra, APCB (Australian Prostate Cancer BioResource), David E. Neal, Sune F. Nielsen, Børge G. Nordestgaard, Henrik Grönberg, Fredrik Wiklund, Robert J. MacInnis, Christopher A. Haiman, Ruth C. Travis, Janet L. Stanford, Adam S. Kibel, Cezary Cybulski, Kay-Tee Khaw, Christiane Maier, Stephen N. Thibodeau, Manuel R. Teixeira, Lisa Cannon-Albright, Hermann Brenner, Radka Kaneva, Hardev Pandha, The PRACTICAL consortium, Jong Y. Park

**Affiliations:** 1 Biostatistics and Data Science Program, School of Public Health, Louisiana State University Health Sciences Center, New Orleans, LA 70112 USA; 2 Information and Communications Research Laboratories, Industrial Technology Research Institute, Hsinchu, Taiwan; 3 The Institute of Cancer Research, London, SM2 5NG UK; 4 Royal Marsden NHS Foundation Trust, London, SW3 6JJ UK; 5 Division of Population Health, Health Services Research and Primary Care, University of Manchester, Oxford Road, Manchester, M13 9PL UK; 6 http://www.icr.ac.uk/our-research/research-divisions/division-of-genetics-and-epidemiology/oncogenetics/research-projects/ukgpcs/ukgpcs-collaborators; 7 Institute of Biomedicine, University of Turku, Turku, Finland; 8 Department of Medical Genetics, Genomics, Laboratory Division, Turku University Hospital, PO Box 52, 20521 Turku, Finland; 9 Department of Applied Health Research, University College London, London, WC1E 7HB UK; 10 Centre for Cancer Genetic Epidemiology, Department of Oncology, University of Cambridge, Strangeways Laboratory, Worts Causeway, Cambridge, CB1 8RN UK; 11 Australian Prostate Cancer Research Centre-Qld, Institute of Health and Biomedical Innovation and School of Biomedical Science, Queensland University of Technology, Brisbane, QLD 4059 Australia; 12 Translational Research Institute, Brisbane, QLD 4102 Australia; 13 Australian Prostate Cancer Research Centre-QLD, Queensland University of Technology, Brisbane, Australia; 14 Prostate Cancer Research Program, Monash University, Melbourne, Australia; 15 Dame Roma Mitchell Cancer Centre, University of Adelaide, Adelaide, Australia; 16 Chris O’Brien Lifehouse and The Kinghorn Cancer Centre, Sydney, Australia; 17 Translational Research Institute, Brisbane, QLD Australia; 18 Nuffield Department of Surgical Sciences, University of Oxford, John Radcliffe Hospital, Room 6603, Level 6, Headley Way, Headington, Oxford, OX3 9DU UK; 19 Department of Oncology, University of Cambridge, Addenbrooke’s Hospital, Hills Road, Box 279, Cambridge, CB2 0QQ UK; 20 Cancer Research UK, Cambridge Research Institute, Li Ka Shing Centre, Cambridge, CB2 0RE UK; 21 Faculty of Health and Medical Sciences, University of Copenhagen, 2200 Copenhagen, Denmark; 22 Department of Clinical Biochemistry, Herlev and Gentofte Hospital, Copenhagen University Hospital, Herlev, 2200 Copenhagen, Denmark; 23 Department of Medical Epidemiology and Biostatistics, Karolinska Institute, 171 77 Stockholm, Sweden; 24 Cancer Epidemiology Division, Cancer Council Victoria, 200 Victoria Parade, East Melbourne, 3002 Australia; 25 Centre for Epidemiology and Biostatistics, Melbourne School of Population and Global Health, The University of Melbourne, Grattan Street, Parkville, VIC 3010 Australia; 26 Center for Genetic Epidemiology, Department of Preventive Medicine, Keck School of Medicine, University of Southern California/Norris Comprehensive Cancer Center, Los Angeles, CA 90015 USA; 27 Cancer Epidemiology Unit, Nuffield Department of Population Health, University of Oxford, Oxford, OX3 7LF UK; 28 Division of Public Health Sciences, Fred Hutchinson Cancer Research Center, Seattle, Washington, 98109-1024 USA; 29 Department of Epidemiology, School of Public Health, University of Washington, Seattle, WA 98195 USA; 30 Division of Urologic Surgery, Brigham and Womens Hospital, 75 Francis Street, Boston, MA 02115 USA; 31 International Hereditary Cancer Center, Department of Genetics and Pathology, Pomeranian Medical University, 70-115 Szczecin, Poland; 32 Clinical Gerontology Unit, University of Cambridge, Cambridge, CB2 2QQ UK; 33 Humangenetik Tuebingen, Paul-Ehrlich-Str 23, 72076 Tuebingen, Germany; 34 Department of Laboratory Medicine and Pathology, Mayo Clinic, Rochester, MN 55905 USA; 35 Department of Laboratory Genetics, Portuguese Oncology Institute of Porto (IPO Porto)/Porto Comprehensive Cancer Center, Porto, Portugal; 36 Cancer Genetics Group, IPO Porto Research Center (CI-IPOP)/RISE@CI-IPOP (Health Research Network), Portuguese Oncology Institute of Porto (IPO Porto)/Porto Comprehensive Cancer Center, Porto, Portugal; 37 School of Medicine and Biomedical Sciences (ICBAS), University of Porto, Porto, Portugal; 38 Division of Epidemiology, Department of Internal Medicine, University of Utah School of Medicine, Salt Lake City, UT 84132 USA; 39 George E. Wahlen Department of Veterans Affairs Medical Center, Salt Lake City, UT 84148 USA; 40 Division of Clinical Epidemiology and Aging Research, German Cancer Research Center (DKFZ), 69120 Heidelberg, Germany; 41 German Cancer Consortium (DKTK), German Cancer Research Center (DKFZ), 69120 Heidelberg, Germany; 42 Division of Preventive Oncology, German Cancer Research Center (DKFZ) and National Center for Tumor Diseases (NCT), Im Neuenheimer Feld 460, 69120 Heidelberg, Germany; 43 Molecular Medicine Center, Department of Medical Chemistry and Biochemistry, Medical University of Sofia, Sofia, 2 Zdrave Str., 1431 Sofia, Bulgaria; 44 The University of Surrey, Guildford, Surrey GU2 7XH UK; 45 Department of Cancer Epidemiology, Moffitt Cancer Center, 12902 Magnolia Drive, Tampa, FL 33612 USA

**Keywords:** SNP interaction, Cluster, False positivity, Simulation, Computational biology and bioinformatics, Genetics, Biomarkers, Risk factors, Mathematics and computing

## Abstract

Single nucleotide polymorphism (SNP) interactions are the key to improving polygenic risk scores. Previous studies reported several significant SNP–SNP interaction pairs that shared a common SNP to form a cluster, but some identified pairs might be false positives. This study aims to identify factors associated with the cluster effect of false positivity and develop strategies to enhance the accuracy of SNP–SNP interactions. The results showed the cluster effect is a major cause of false-positive findings of SNP–SNP interactions. This cluster effect is due to high correlations between a causal pair and null pairs in a cluster. The clusters with a hub SNP with a significant main effect and a large minor allele frequency (MAF) tended to have a higher false-positive rate. In addition, peripheral null SNPs in a cluster with a small MAF tended to enhance false positivity. We also demonstrated that using the modified significance criterion based on the 3 p-value rules and the bootstrap approach (3pRule + bootstrap) can reduce false positivity and maintain high true positivity. In addition, our results also showed that a pair without a significant main effect tends to have weak or no interaction. This study identified the cluster effect and suggested using the 3pRule + bootstrap approach to enhance SNP–SNP interaction detection accuracy.

## Introduction

In the past decade, inherited genetic variant or single nucleotide polymorphism (SNP) data generated from Genome-Wide Association Studies (GWAS) increased dramatically because of the decreasing cost of genotyping arrays, increasing number of testing variants in arrays, increasing interest in new phenotypes (such as treatment effects), and development of advanced statistical analyses^1,2^. Most GWAS-identified SNPs can only provide a small prediction individually. Recently, many polygenic risk scores (PRSs), the weighted sum of risk variants based on SNP main effects, for various phenotypes have been proposed^3^. PRS can provide a score to quantify an individual’s genetic risk, and these polygenic risk scores benefit personalized medicine. The polygenic risk scores have been shown to increase prediction power for complex traits compared with a single SNP, but there is room for improvement. Most PRSs do not consider SNP–SNP interactions. It has been established that gene–gene/SNP–SNP interactions play a more prominent role in the causality of complex diseases^4,5^. It has been shown that analyses of SNP–SNP interactions or epistasis are important post-GWAS and potential solutions for solving missing heritability^2^.

Although SNP–SNP interactions have received more attention in the past decade, many SNP–SNP interaction studies suffer low statistical power due to inappropriate statistical methods. Many SNP–SNP interactions have been identified, but few can be replicated. One of the reasons is the use of the conventional statistical method, the Additive-Additive full interaction (AA-Full) method, for testing SNP–SNP interactions. This AA-Full method tests the full or hierarchical interaction model (2 SNPs + their interaction), and each SNP is based on additive inherited mode (count of minor alleles as 0, 1, and 2). It has been shown that AA-Full has low power for detecting SNP–SNP interactions and tends to lead to false negative results because this approach only tested one complicated interaction pattern^6,7^. Sufficient statistical power is critical for successful investigations, and studies with low statistical power result in false negativity, which wasted research resources^8^.

A SNP-interaction cluster is defined as a set of SNP–SNP interaction pairs sharing a common or hub SNP. When advanced statistical methods were used, SNP-interaction clusters have been reported in many studies. More SNP–SNP interactions have been identified with the development of advanced and powerful statistical methods for evaluating SNP–SNP interactions, but many related features remain unclear. We observed that an increasing number of published studies showed that many significant SNP–SNP interaction pairs are grouped into a SNP-interaction cluster^9–14^, which refers to a set of SNP–SNP interaction pairs sharing a common or hub SNP. Even though these SNP–SNP interaction studies used different statistical methods for various phenotype outcomes, the cluster effect for significant SNP–SNP interactions has been reported^9,10,13,14^. For example, our previous study evaluated SNP–SNP interactions associated with prostate cancer aggressiveness and identified 4 SNP-interaction clusters using the 2-stage AA9int-SIPI approach^9^. A study tested high-order SNP–SNP interactions associated with age-related macular degeneration using the multi-population harmony search algorithm, an artificial intelligence approach^10^. Using this new approach, 169 SNP pairs were in 3 clusters with a size of 138, 24, and 7 pairs, respectively. Moreover, one SNP was shown in all 3- and 4-order SNP–SNP interactions, and another SNP was included in all 4-order interactions^10^. The other 3 studies using the harmony search algorithm also showed the cluster effect of significant SNP–SNP interactions^15–17^. Another study evaluated SNP–SNP interactions associated with rheumatoid arthritis. There are 19 out of the top 20 SNP–SNP interaction pairs in the 2 clusters (1 cluster with 6 pairs and the other with 13 pairs) based on three non-parametric methods^13^. Moreover, studies using the Multifactor Dimensionality Reduction (MDR) method, a model-free data mining method for detecting SNP–SNP interactions, showed that SNPs with a strong main effect increase the chance of significant SNP–SNP interactions^18–23^. These studies showed that many hub SNPs in the SNP-interaction clusters also had significant main effects. Thus, we hypothesize that some pairs in the cluster of significant SNP–SNP interaction pairs are false positive, and the false positivity is related to the significance level of the hub SNPs’ main effect.

To apply identified SNP–SNP interactions for risk prediction and biological mechanisms, it is essential to understand the false positivity issue of SNP–SNP interactions and develop a tool to reduce false positivity. The bootstrap, a resampling technique, has been used for estimating statistics, statistical tests, and variable selection, and in SNP association studies, it is used to distinguish true positives from false positives^24^. However, the usage and performance of the bootstrap method for SNP–SNP interaction are still understudied. Therefore, this study aims to evaluate cluster effect features of significant SNP–SNP interaction pairs, identify factors associated with false positivity, and develop methods for improving SNP–SNP interaction detection accuracy.

## Methods

**Figure 1:**
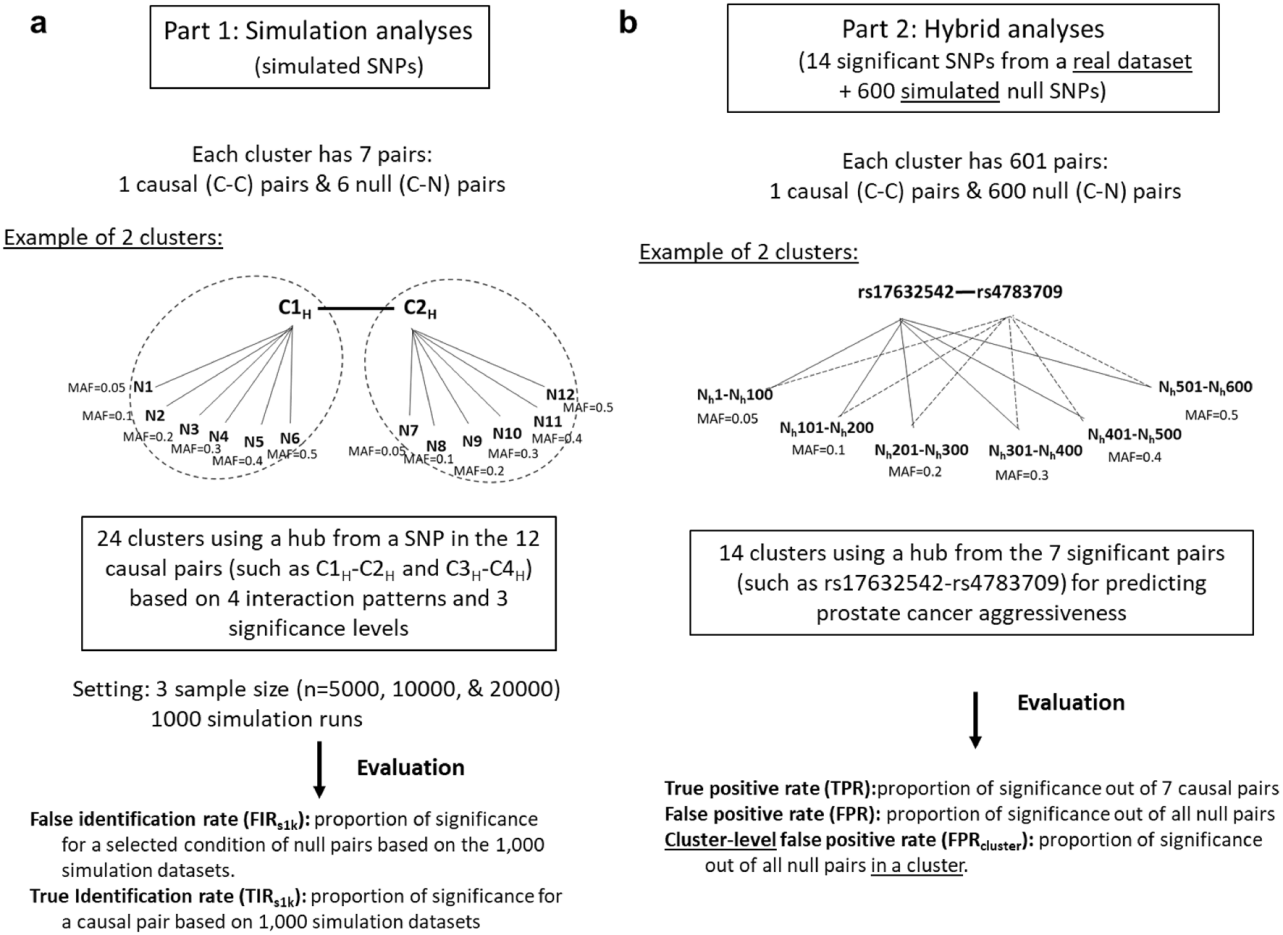
Summary of study design in the 2 study parts. (**a**) Simulation setting in Part 1: A cluster with 7 pairs: one causal (C–C) pair, and 6 with null (C–N) pairs. (**b**) Hybrid setting in Part 2: A cluster with 601 pairs: one observed causal pair and 600 simulated null pairs. *MAF* minor allele frequency, “C” represents a SNP from a causal pair; “N” represents a simulated null SNP.

We are interested in evaluating factors associated with false- and true-positivity for SNP–SNP interaction analyses using the SNP Interaction Pattern Identifier (SIPI) approach^6^, focusing on SNP-interaction pairs in a cluster. A SNP-interaction cluster is defined as a set of SNP–SNP interaction pairs sharing a common or hub SNP. For thoroughly evaluating false-positivity and true-positivity for SNP–SNP interactions, this study has 2 parts (Fig. 1a,b). Part 1 is based on simulation analyses with 1000 simulation runs (or 1000 simulated datasets) for each condition. Part 2 is to mimic real data analyses based on one dataset using a hybrid study with both observed and simulated data. In this study, we used “C” to denote a SNP in a causal pair, which was associated with the outcome. “N” represented a null SNP generated based on simulation that was not associated with the outcome. Thus, C–C pairs are causal pairs, and C–N and N–N pairs are null pairs, which are not significantly associated with the outcome.

### Method of testing SNP main effects

For SNP main effects, various inheritance modes (dominant, recessive, and additive) were assessed using logistic regressions for a binary outcome. The best mode was selected based on the lowest p-value for each SNP using the “SNPmain” function in the SIPI R package. Our published study shows that SNP inheritance modes play an essential role in association tests because the additive model assumption may not be valid for all conditions^25^.

### Method of testing SNP–SNP interactions

We tested SNP–SNP interactions associated with an outcome using the SNP Interaction Pattern Identifier (SIPI) approach^6^. With a binary outcome, logistic-model-based SIPI was applied. The analyses used the “SIPI” function in the SIPI R package. For each SNP pair, SIPI tests 45 interaction patterns by considering 3 major factors: model structures, inheritance modes, and risk directions. There are 4 model structures: (1) full interaction model with two SNPs plus the interaction of SNP1 and SNP2; (2) SNP1 plus an interaction; (3) SNP2 plus an interaction; and (4) interaction only. The 3 inheritance modes are additive, dominant, and recessive modes. In addition, SIPI considers 2 risk or mode-coding directions: original coding based on a minor allele and reverse coding. SIPI uses the Bayesian information criterion to search for the best interaction pattern with the smallest Bayesian information criterion^6^. Based on our previous studies^6,9^, the most common models for the significant SNP–SNP interaction pairs are interaction-only models. For p-values, “p-main” is defined as the p-value of a SNP main effect associated with an outcome in a model with this SNP main effect only without interaction, and “p-pair” is defined as a p-value of a SNP–SNP interaction pair associated with an outcome.

In conventional studies, researchers often apply a p-value cut-point (called “p-pair-criterion,” such as the Bonferroni criterion) to identify significant SNP–SNP interaction pairs. Based on the results of previous studies^9,10^ and Table 1, SNP–SNP interaction pairs with a significant SNP main effect tend to have a significant interaction. In addition, most of the research interest is identifying SNP–SNP interactions, which can predict the outcome better than SNP's main effects. Thus, we proposed “3pRule”, a modified significance rule for detecting SNP–SNP interactions. The “3pRule” approach is a rule of defining a significant SNP interaction pair based on 3 p-value rules: (1) p-pair of SNP1-SNP2 < p-pair-criterion; (2) p-pair < p-main of SNP1, and (3) p-pair < p-main of SNP2. Then, we compared the performance of 3pRule with the convention approach (called “1pRule”). The “1pRule” approach is a convention rule of defining a significant SNP interaction pair based on 1 p-value criterion: p-pair of SNP1-SNP2 < p-pair-criterion. It is worth mentioning that the 1pRule and 3pRule approaches have different results only when p-SNP1 and/or p-SNP2 are less than the p-pair criterion, which indicates that these SNPs have a very significant main effect, especially GWAS-identified SNPs.

**Table 1:** Summary of 7 observed significant SNP–SNP interaction pairs associated with a binary outcome.

SNP pair SNP1–SNP2	SNP1 Min < Maj (MAF)^1^	SNP2 Min < Maj (MAF)^1^	SNP1 p-value (p-main)^2^	SNP2 p-value (p-main)^2^	SNP–SNP interaction p-value (p-pair)^2^
rs17632542-rs4783709	G < A (0.06)	A < G (0.31)	**2.2 × 10**^**–15**^	0.027	**5.7 × 10**^**–18**^
rs2569735-rs7613553	A < G (0.12)	A < C (0.44)	**5.5 × 10**^**–9**^	0.551	**4.4 × 10**^**–13**^
rs1058205-rs2274545	G < A (0.15)	C < A (0.28)	**9.5 × 10**^**–8**^	0.065	**8.5 × 10**^**–10**^
rs4802755-rs4473378	A < G (0.46)	G < A (0.14)	**1.8 × 10**^**–7**^	0.728	**2.3 × 10**^**–9**^
rs174776-rs1250240	A < G (0.11)	A < G (0.26)	**7.9 × 10**^**–7**^	0.279	**3.5 × 10**^**–10**^
rs2271095-rs7446	G < A (0.35)	A < G (0.31)	**2.0 × 10**^**–6**^	**2.0 × 10**^**–5**^	**1.7 × 10**^**–12**^
rs266876-rs9521694	G < A (0.24)	A < T (0.16)	**3.2 × 10**^**–6**^	0.001	**3.4 × 10**^**–9**^

^1^*Min* minor allele, *Maj* major allele, *MAF* minor allele frequency.

^2^Bold for significant results based on the Bonferroni criteria. p-main < 8.1 × 10^–5^ (= 0.05/614), and p-pair < 2.7 × 10^–7^ (= 0.05/^614^C_2_).

### Part 1: simulation analyses

**Figure 2:**
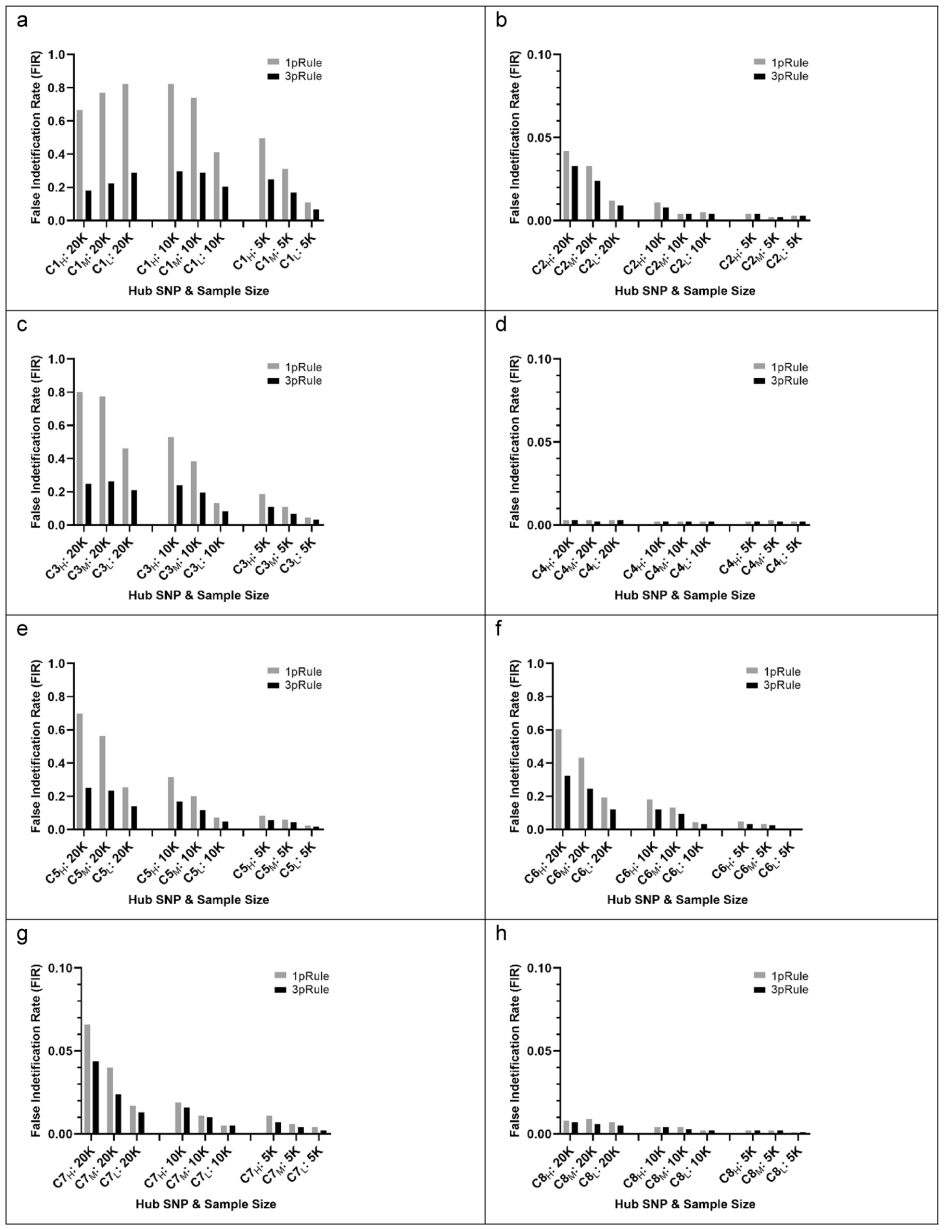
False identification rates (FIR_s1k_) for the 8 sets of clusters based on 1000 simulation runs. Each set had 3 clusters with a hub SNP with various significance levels, such as C1_H_, C1_M_, and C1_L_ for C1 SNP with a high, medium, and low significance level, respectively. Sample size: 20 K (n = 20,000), 10 K (n = 10,000), and 5 K (n = 5000). Significance rules: 1pRule: p-pair < 2.7 × 10^–7^; 3pRule: p-pair < 2.7 × 10^–7^ and p-pair < p-main for SNP1, and p-pair < p-main for SNP2.

In the simulation in Part 1, we evaluated the cluster effect of significant SNP–SNP interactions, focusing on positivity in a cluster. In Part 1, we evaluated 24 clusters with 7 pairs per cluster (1 causal (C–C) pair and 6 null (C–N) pairs, see Fig. 1a). The hub SPNs of these 24 clusters were based on the 24 SNPs in the 12 causal pairs associated with a binary outcome. Under a similar interaction pattern, we were interested in evaluating the false positivity of pairs with different p-pair and p-main for the hub SNP in a cluster. Therefore, these 12 causal pairs were generated based on 4 interaction patterns with 3 various significance levels (low significance (L), medium significance (M), and high significance (H)) (Supplementary Table S1 and Fig. 2). In order to mimic complicated relationships of SNP–SNP interactions associated with a binary outcome, the 4 pairs with a high significance level (C1_H_–C2_H_, C3_H_–C4_H_, C5_H_–C6_H_, and C7_H_–C8_H_) were generated the top findings of our published study with a sample size of around 20,000^9^. As shown in Supplementary Table S1, the 4 SNP pairs with a high significance level had a p-pair of 4.5 × 10^–18^ to 6.7 × 10^–5^ under a sample size of 20,000 and had a wide range of MAF (0.055–0.444). Then, the other 8 pairs were generated using the same 4 interaction patterns but with lower significance levels. The 4 pairs with a medium significance level were C1_M_–C2_M_, C3_M_–C4_M_, C5_M_–C6_M_, and C7_M_–C8_M_, and the 4 pairs with a low significance level of C1_L_–C2_L_, C3_L_–C4_L_, C5_L_–C6_L_, and C7_L_–C8_L_. Therefore, 3 pairs for each set were generated. Using the C1–C2 set as an example, C1_H_–C2_H_, C1_M_–C2_M_, andC1_L_–C2_L_ are the pairs with the similar C1–C2 pattern with the high, medium, and low significance of the SNP–SNP interaction (p-pair = 4.5 × 10^–18^, 9.1 × 10^–14^, and 1.6 × 10^–8^, respectively) under a sample size of 20,000. The details of simulating these causal pairs are listed in the Supplementary Methods S1 section.

For null pairs in a cluster (C–N pairs), we simulated 6 null SNPs independently based on various MAFs of 0.05, 0.1, 0.2, 0.3, 0.4, and 0.5, named N1 to N6. The null SNPs were generated based on the MAF following a multinomial distribution, with the probabilities based on the Hardy–Weinberg equilibrium (HWE). Because these SNPs were generated independently, they were not associated with the outcome. Thus, each cluster had 7 pairs (see Fig. 1a). Using the cluster with C1_H_ as the hub SNP as an example, there are 1 causal pair (such as C1_H_–C2_H_) and 6 null pairs (including C1_H_–N1, C1_H_–N2 to C1_H_–N6). Each condition was tested under 3 sample sizes (n = 5000, 10,000, and 20,000) and 1000 simulation runs. For evaluation, both 1pRule and 3pRule with a p-pair-criterion less than 1 × 10^–4^ based on empirical results (Supplementary Fig. S2b) were applied to detect significant pairs. TIR_s1k_ and FIR_s1k_ were used to measure the probability of being significant based on 1000 simulation datasets under a selected condition. “True identification rate (TIR_s1k_)” is defined as a proportion of significance for a causal pair out of the 1000 simulation datasets. “False identification rate (FIR_s1k_)” was defined as a proportion of significance for a selected condition of null pairs (such as the 6 null pairs with C1_H_ as a hub under a sample size of 20,000) based on the 1000 simulation datasets.

### Part 2: hybrid study

In order to develop methods for improving SNP–SNP interaction detection accuracy and evaluate N–N pairs, we applied a hybrid study by integrating causal pairs from real data and simulated null SNPs. As shown in Table 1 and Fig. 1b, this hybrid study used a dataset comprising 614 SNPs: 14 SNPs from 7 significant pairs and 600 null SNPs. We evaluated 14 clusters, with each of these 14 SNPs as a hub SNP. For causal pairs, we included 7 observed SNP–SNP interaction pairs related to the *KLK3* gene significantly associated with prostate cancer aggressiveness based on our published study and randomly selected 20,000 subjects from the original data^9^. These 7 pairs were treated as causal pairs with true associations. These 7 SNP–SNP interaction pairs are rs17632542-rs4783709 in *KLK3* and *CYB5B:LOC105371325* and rs2569735-rs7613553 in *KLK3* and *RARB*, rs1058205-rs2274545 in *KLK3* and *COL4A2*, rs4802755-rs4473378 in *KLK3* and *FN1-DT*, rs174776-rs1250240 in *KLK3* and *FN1*, rs2271095-rs7446 in *KLK3* and *KPNA3*, rs266876-rs9521694 in *KLK3* and *COL4A2* associated with prostate cancer aggressiveness with a p-value of interactions in a range of 5.7 × 10^–18^ to 3.4 × 10^–9^. The p-pair values of 7 C–C pairs and the p-main values of their composite SNPs associated with prostate cancer aggressiveness are listed in Table 1. The MAF of these 14 SNPs in the causal pairs ranged from 0.06 to 0.46. For significance, the Bonferroni criterion p < 8.1 × 10^–5^ (= 0.05/614) was applied for SNP main effects, and a p-pair-criterion of 2.7 × 10^–7^ (= 0.05/^614^C_2_) was used for SNP–SNP interaction pairs for both 1pRule and 3pRule.

For null SNPs, we simulated 600 null SNPs (N1, N2, …, N600) independently based on the HWE with a sample size of 20,000. We generated 100 null SNPs for the 6 different MAF conditions (0.05, 0.1, 0.2, 0.3, 0.4, and 0.5). Among all pairs, we were especially interested in evaluating the clusters with 1 causal pair and 600 null pairs (C–N pairs) in the same cluster (Fig. 1b). For a total of 614 SNPs, there were 188,191 pairwise interaction pairs: 7 causal C–C pairs, 8400 C–N pairs, 179,700 N–N pairs, and 84 pairs with other combinations of 2 “C” SNPs (called “C–C-other” pairs). We defined the 7 selected significant pairs as causal pairs and the others as null pairs. Therefore, any observed significant C–N, N–N, or C–C-other pairs in this study were considered false positive findings. For testing correlations or the LD status among SNPs, r^2^ was applied. SNPs with r^2^ > 0.8 were considered strong LD. For the hybrid project in Part 2, the true positive rate (TPR) was defined as a proportion of significance out of 7 causal pairs. The false positive rate (FPR) was defined as a proportion of significance out of all null pairs. In addition, the cluster-level FPRs (FPR_cluster_) was defined as a proportion of significance out of null pairs in a cluster.

In the 2nd part of the hybrid analysis, we were also interested in evaluating correlations between a C–C pair and the significant corresponding C–N pairs in the same cluster. All 7 causal pairs and most significant null pairs had interaction-only patterns analyzed by SIPI. For pairs with an interaction-only pattern with an additive mode, these pairs with a value of (0, 1, 2, or 4) can be treated as a continuous variable. Thus, Pearson correlations can be used to calculate correlations between these pairs with an additive mode. The Phi correlation was calculated for the interaction patterns with binary (0 and 1) dominant or recessive inheritance modes. Moreover, we further tested correlations between p-pair and p-main, the most significant main effect in the 2 composite SNPs, using the Spearman test for 91 pairwise interactions based on the 14 SNPs in the causal pairs.

### Bootstrap variable selection

Based on our study findings, 3pRule can effectively reduce FPRs compared with 1pRule. However, we observed that some false positive pairs were highly correlated with the causal pairs even after applying 3pRule. In order to solve this issue, we proposed using the “bootstrap + 3pRule” approach and applied it in Part 2. In the bootstrap approach or resampling with replacement, subjects are randomly selected with replacement, miming the sampling variation in the population from which the sample was drawn^26^. The sample size of bootstrap datasets was the same as the observed data. In order to mimic real data analyses, the 500 bootstrap samples were generated based on the observed dataset with 614 SNPs and the same sample size of 20,000. On each bootstrap dataset, we performed pairwise SNP–SNP interaction for the 614 SNPs using SIPI, in which 3pRule was applied to define significant pairs. For evaluating the performance of the “3pRule + bootstrap” approach, the positivities (TPR and FPR) were compared with the conventional approach, original data with 1 pRule, based on 500 bootstrap datasets.

The SIPI function in the SIPI R package (https://github.com/LinHuiyi/SIPI) was used to detect SNP–SNP interaction pairs. In addition, the 3 new functions have been added to the SIPI package based on the findings of this study. The “*eval3pRule”* R function is to identify significant SNP–SNP interaction pairs based on 3pRule. The “boot3p_SIPI” R function is used to conduct SIPI analyses to detect SNP–SNP interactions with the “bootstrap + 3pRule” approach. Using this function, the SIPI results based on the 3pRule in the user-defined bootstrap datasets will be summarized. The “*bootData”* R function is for bootstrap data generation.

## Results

For Part 1, we are interested in evaluating FIR_s1k_ for pairs in a cluster. As shown in Fig. 2a–h and Supplementary Table S1, FIR_s1k_ based on 1pRule were larger than 3pRule. For C1 with a high significance level (p-main = 1.4 × 10^–8^) under a sample size of 10,000, the FIR_s1k_ was 82.3% for 1pRule but reduced to 29.5% for using 3pRule. For C3 with a high significance level (p-main = 2.1 × 10^–7^) under a sample size 10,000, the FIR_s1k_ was 53.0% for 1pRule but reduced to 23.8% for using 3pRule. These results support that 3pRule can effectively reduce FIR_s1k_ compared with the 1pRule. As for the sample size effect, we observed that a large sample size caused a high FIR_s1k_, and this trend was applied for both 1pRule and 3pRule. For example, FIR_s1k_ for C3 with a high significance level were 24.8%, 23.8%, and 10.8% under a sample size of 20,000, 10,000, and 5000, respectively, based on 3pRule. In addition, the significance level of the main effect (p-main) for the hub SNP also affected FIR_s1k_. The smaller value of the hub SNP’s p-main generally had a higher FIR_s1k_. Using the C1 cluster as an example (Fig. 2a), the FIR_s1k_ were 29.5%, 28.8%, and 20.5% of C1 with p-main of 7.5 × 10^–10^, 9.6 × 10^–8^, and 3.8 × 10^–5^, respectively, under a sample size of 10,000. For the C3-cluster under the sample size 10,000 (Fig. 2c), the FIR_s1k_ were 23.8%, 19.5%, and 8.2% for C3 with p-main of 2.1 × 10^–7^, 5.0 × 10^–6^, and 4.1 × 10^–4^, respectively. A similar FIR_s1k_ trend can be observed in other clusters, as shown in Fig. 2.

**Figure 3:**
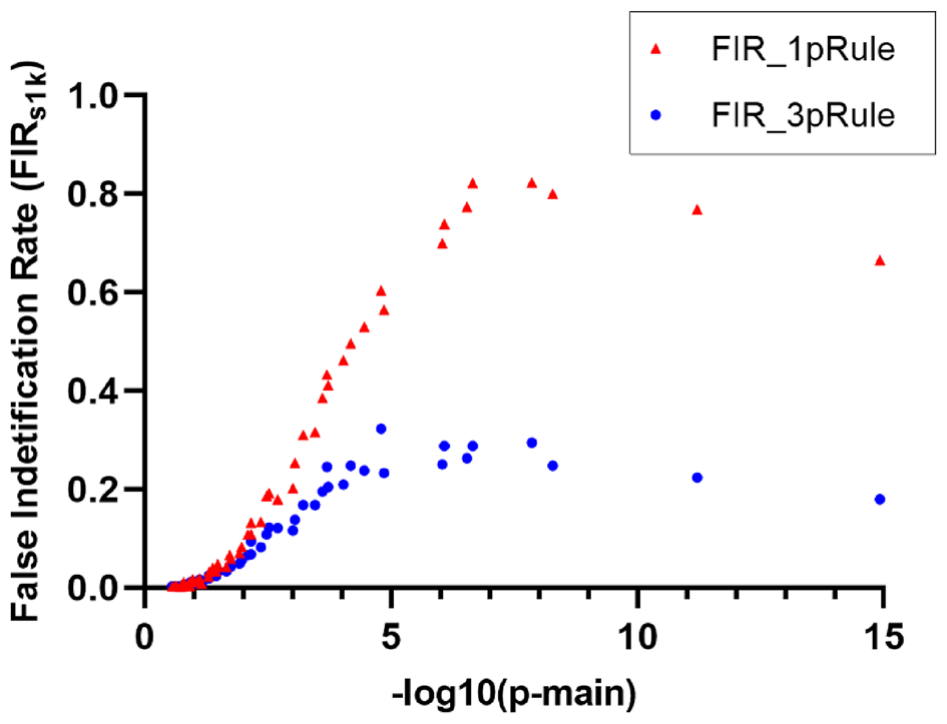
False identification rates (FIR_s1k_) by the hub SNP’s main effect (p-main) and the 2 significance rules. Results were based on the 1000 simulation runs of the 72 clusters and their hub SNPs. Two Significance rules: 1pRule: p-pair < 2.7 × 10^–7^ ; 3pRule: p-pair < 2.7 × 10^–7^ and p-pair < p-main for SNP1, and p-pair < p-main for SNP2.

**Figure 4:**
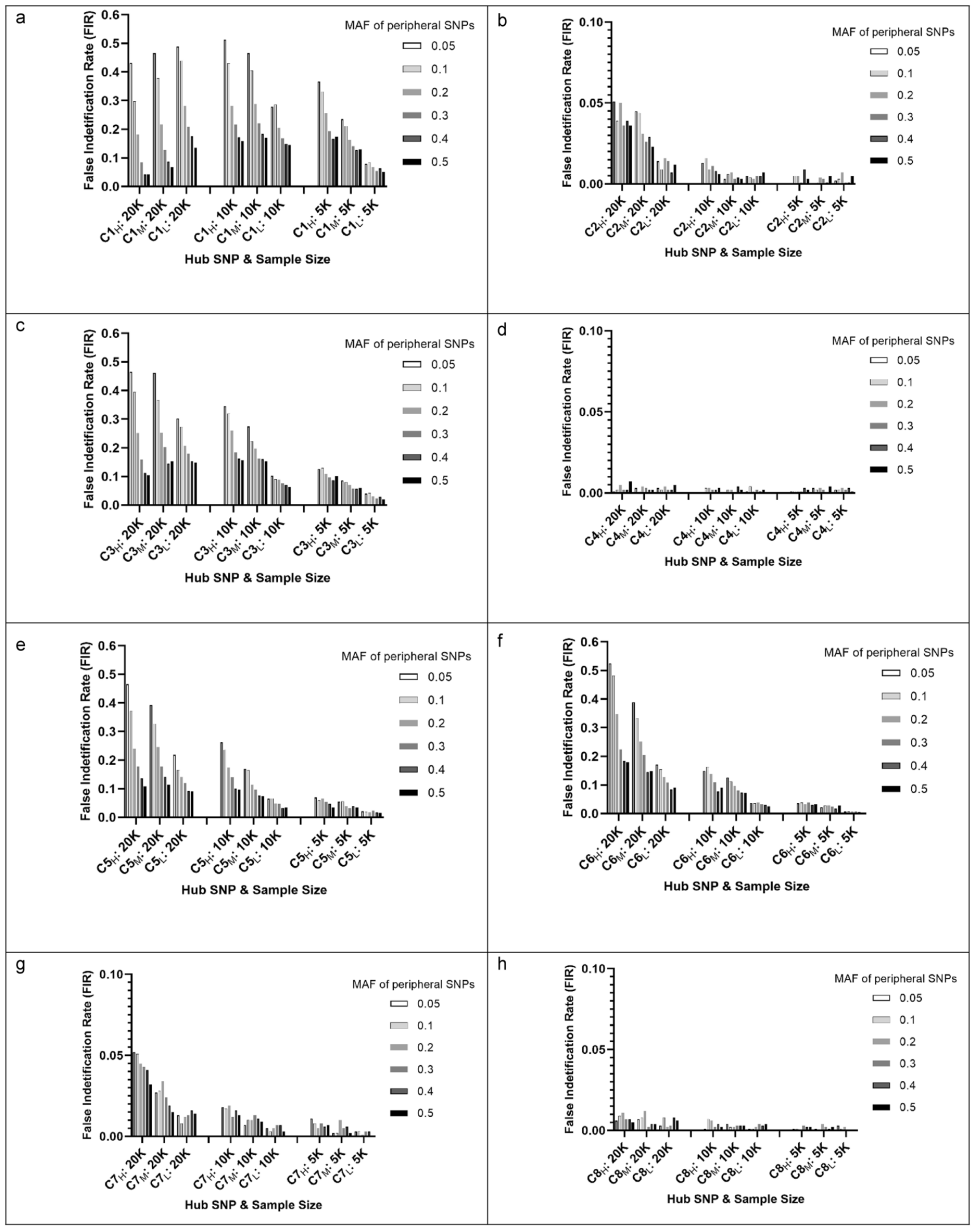
False identification rates (FIR_s1k_) for 8 sets of SNP–SNP interaction clusters based on 3pRule and 1000 runs. Each set had 3 clusters with a hub SNP with various significance levels, such as C1_H_, C1_M_, and C1_L_ for C1 SNP with a high, medium, and low significance level, respectively. Sample size: 20 K (n = 20,000), 10 K (n = 10,000), and 5 K (n = 5000).

All FIR_s1k_ results listed in Supplementary Table S1 were summarized in Fig. 3 with 72 FIR_s1k_ results by the hub SNP’s p-main and the 2 significance rules. Each data point represented the results of a cluster with 6 null pairs (such as C1_H_–N1, C1_H_–N2, and C1_H_–N6). The FIR_s1k_ of null SNPs in a cluster were positively associated with p-main values of the hub SNP. The smaller values of p-main, which equals the larger value of − log10 (p-main), the higher the FIR_s1k_, and the 3pRule approach can reduce FIR_s1k_ compared with the conventional 1pRule. Moreover, FIR_s1k_ were also affected by the MAF status of the peripheral SNPs. As shown in Fig. 4, peripheral SNPs with a low MAF tended to have higher FIR_s1k_ than those with a large MAF. Using the C1_H_ (C1 with high significance, p-main = 1.4 × 10^–8^) under a sample size of 10,000 as an example, the FIR_s1k_ for peripheral SNPs with MAF values of 0.05, 0.1, 0.2, 0.3, 0.4, and 0.5 were 51.2%, 43.0%, 28.2%, 21.6%, 17.2% and 15.8%, respectively. This means that C1_H_ with peripheral SNPs with a 0.05 MAF had 51.2% chance of being false positive, and the false positive chance was reduced to 15.8% when peripheral SNPs with a large MAF of 0.5. Similar trends can be observed for other conditions (Fig. 4).

**Figure 5:**
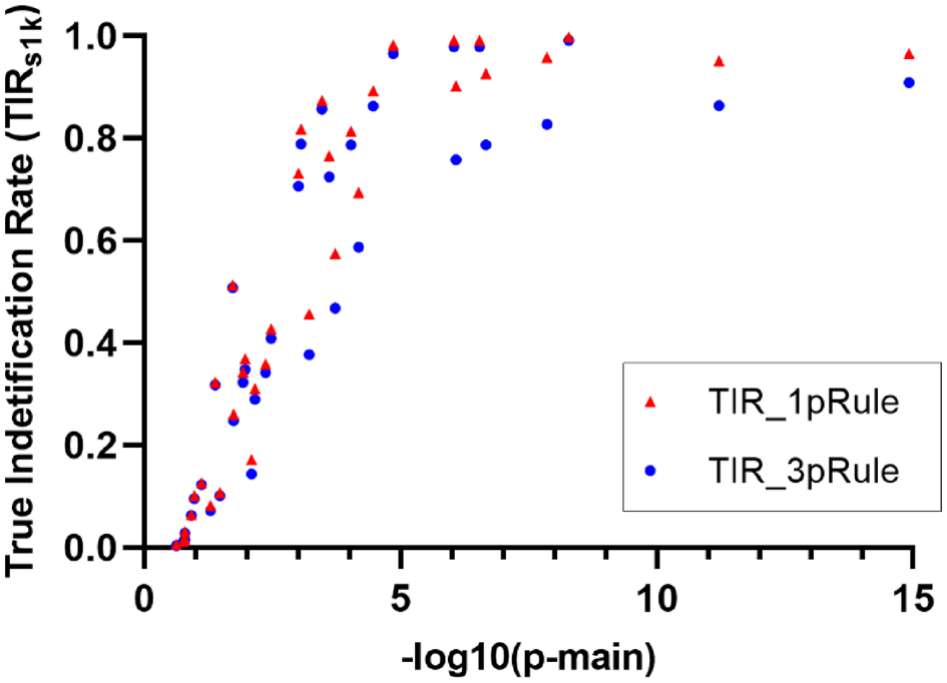
True identification rates (TIR_s1k_) by the hub SNP’s main effect (p-main) and the 2 significance rules. In this plot, the most significant SNP in a causal pair was used as a hub. Results were based on the 1000 simulation runs of the 36 clusters and their hub SNPs. Two Significance rules: 1pRule: p-pair < 2.7 × 10^–7^; 3pRule: p-pair < 2.7 × 10^–7^ and p-pair < p-main for SNP1, and p-pair < p-main for SNP2.

For Part 1, the TIR_S1K_ for the 12 pairs under the 3 sample sizes (n = 20,000, 10,000, and 5000) based on 1000 simulation runs are listed in Supplementary Fig. S1. As shown in Fig. 5 and Supplementary Fig. S1, the TIR_S1K_ for 3pRule was lower but similar to those for 1pRule under the same condition in general. The 3pRule has more stringent criteria to define significance than the 1pRule. Therefore, we can expect that the TIR_S1K_ for 3pRule is lower than for 1pRule. For example, the TIR_S1K_ for the C1–C2 pair with the highly significant interaction (p-pair = 4.5 × 10^–18^) were 96.5% vs. 90.9% based on 1000 simulation runs by using 1pRule vs. 3pRule, respectively, under a sample size of 20,000. For the C3–C4 pairs with a highly significant interaction (p-pair = 3.9 × 10^–13^), their TIR_S1K_ were 99.8% vs. 99.1% using 1pRule vs. 3pRule, respectively, under a sample size of 20,000. For the effect of sample size, TIR_S1K_ was higher for a large sample size. For example, the TIR_S1K_ for the C1–C2 pair with a high significance of interaction were 90.9%, 82.7%, and 58.7% by using 3pRule under a sample size of 20,000, 10,000, and 5000, respectively. As expected, the significance level of the interaction also decreased as the sample size decreased. For example, the p-pair values for the C1–C2 pair with a high significance of interaction were 4.5 × 10^–18^, 7.5 × 10^–10^, and 9.0 × 10^–6^ under a sample size of 20,000, 10,000, and 5000, respectively (Supplementary Table S1). We were interested in further evaluating the relationship between TIR_S1K_ for causal pairs and the p-main of their most significant composite SNP. Furthermore, all TIR_s1k_ results for the 36 conditions by the 2 significance rules were summarized in Fig. 5. Each data point represented the results of a causal pair (such as C1_H_–C2_H_). Figure 5 shows a positive relationship between TIR_S1K_ and the p-main values of the most significant composite SNP. In addition, the TIRs for 3pRule are lower but similar to the TIR_S1K_ of 1pRule. In summary of Part 1, 3pRule can effectively reduce FIR_s1k_ and maintain TIR_S1K_ compared to 1pRule for detecting SNP–SNP interactions.

### Part 2: hybrid study

For the dataset of 614 SNPs in Part 2, 7 causal pairs (C–C pairs) are only 0.004% of the total 188,191 pairs, so identifying these 7 causal pairs and keeping low FPRs is a challenge. All 7 SNP pairs were significant based on the Bonferroni criterion (p-pair < 2.7 × 10^–7^), and the range of their p-pair value was 5.7 × 10^–18^ (rs17632542–rs4783709) to 3.4 × 10^–9^ (rs266876–rs9521694). Interestingly, each causal pair had at least 1 SNP with a significant main effect. Table 1 showed that SNP pairs with a low p-main of the composite SNP tended to be more significantly associated with the outcome. For further testing, we tested correlations between the p-pair and p-main values for the powerful SIPI approach and the conventional AA-full model approach. Among the 91 pairwise interactions based on the 14 observed SNPs, a significant positive correlation was observed between the p-pair and p-main of the most significant composite SNP for the SIPI approach (Spearman r = 0.73, p < 0.001) but not for the AA-full approach (Spearman r = 0.17, p = 0.118). This demonstrated that the high correlations between p-pair and corresponding p-main values only exist in the SIPI but not in the low-power AA-full approach.

Among the 14 clusters with a hub SNP involved in the 7 C–C pairs, FPR_cluster_ were 0% for the 6 SNPs with a p-main ≥ 1 × 10^–4^ and various MAFs of 0.14–0.44. For the remaining 8 SNPs with a p-main < 1 × 10^–4^, the FPR_cluster_ for these 8 clusters were summarized in Table 2. The following FPR_cluster_ discussions are primarily based on 3pRule. We observed that the pairs with a hub SNP with an insignificant main effect had 0% FPR_cluster_ despite the hub SNP’s MAF. The clusters tended to have a high FPR_cluster_ if the hub SNP had a significant p-main and a large MAF. Mainly, rs4802755 had a significant effect (p-main = 1.8 × 10^–7^) with a large MAF (0.46), so its cluster yielded the highest FPR_cluster_ (38.7%) compared with other clusters. In contrast, rs17632542 had the most significant main effect (p-main = 2.2 × 10^–15^) but had a low MAF of 0.06. Therefore, its FPR_cluster_ is 15.0%, which is lower than the FPR_cluster_ of the rs4802755 cluster. For the pair of rs2271095-rs7446, both SNPs had a significant main effect. rs7446 and rs2271095 had similar MAFs of 35% and 31%, respectively. rs2271095 (p-main = 2.0 × 10^–6^) had a more significant main effect than rs7446 (p-main = 2.0 × 10^–5^), so rs2271095 had a higher FPR_cluster_ than rs7446 (FPR_cluster_ = 2% vs. 0.3%). In addition, these cluster effects could be observed in Supplementary Fig. S2a with 4 obvious clusters with a small p-pair. The most significant cluster is the rs17632542 cluster, followed by rs2569735, which had the same order of their p-pair and p-main values.

**Table 2:** Cluster-level false positive rates (FPR_cluster_) in a SNP-pair cluster by 2 significance rules and minor allele frequency (MAF) of peripheral null SNPs.

Hub SNP (causal pair)	p-main of hub (p-pair)	MAF of hub	1pRule	3pRule	3pRule					
			Total	Total	Set-1 MAF = 0.05	Set-2 MAF = 0.1	Set-3 MAF = 0.2	Set-4 MAF = 0.3	Set-5 MAF = 0.4	Set-6 MAF = 0.5
			FPR_cluster_% of 600 pairs^1^	FPR_cluster_% of 600 pairs (corr)^1^	FPR_cluster_% of 100 pairs (corr)^1^	FPR_cluster_% of 100 pairs (corr)^1^	FPR_cluster_% of 100 pairs (corr)^1^	FPR_cluster_% of 100 pairs (corr)^1^	FPR_cluster_% of 100 pairs (corr)^1^	FPR_cluster_% of 100 pairs (corr)^1^
rs17632542 (rs17632542-rs4783709)	2.2 × 10^–15^ (5.7 × 10^–18^)	0.06	66.5	15.0 (0.86)	34 (0.88)	21 (0.86)	26 (0.85)	6 (0.82)	2 (0.77)	1 (0.72)
rs2569735 (rs2569735-rs7613553)	5.5 × 10^–9^ (4.4 × 10^–13^)	0.12	79.5	19.2 (0.74)	32 (0.78)	24 (0.77)	33 (0.74)	10 (0.69)	8 (0.64)	8 (0.64)
rs1058205 (rs1058205-rs2274545)	9.5 × 10^–8^ (2.0 × 10^–9^)	0.15	39.8	18.5 (0.80)	39 (0.85)	26 (0.81)	22 (0.82)	7 (0.75)	11 (0.68)	6 (0.64)
rs4802755 (rs4802755-rs4473378)	1.8 × 10^–7^ (2.3 × 10^–9^)	0.46	48.5	38.7 (0.77)	80 (0.80)	61 (0.79)	40 (0.77)	26 (0.73)	19 (0.69)	6 (0.64)
rs174776 (rs174776-rs1250240)	7.9 × 10^–7^ (2.4 × 10^–9^)	0.11	7.8	7.8 (0.29)	7 (0.31)	9 (0.32)	12 (0.31)	9 (0.27)	7 (0.30)	3 (0.18)
rs2271095 (rs2271095-rs7446)	2.0 × 10^–6^ (1.7 × 10^–12^)	0.31	2.0	2.0 (0.48)	2 (0.53)	3 (0.51)	3 (0.46) 0 -	0 -	4 (0.45)	0 -
rs7446 (rs2271095-rs7446)	2.0 × 10^–5^ (1.7 × 10^–12^)	0.35	0.3	0.3 (0.36)	0 -	1 (0.42)		1 (0.30)	0 -	0 -
rs266876 (rs266876-rs9521694)	3.2 × 10^–6^ (3.4 × 10^–9^)	0.24	2.3	2.3 (0.61)	2 (0.72)	4 (0.67)	4 (0.59)	2 (0.49)	3 (0.58)	0 -

^1^FPR_cluster_% and mean of correlations (corr), calculated between the causal pair and significant null pairs sharing the same hub SNP in a cluster (such as rs17632542-rs4783709 pair correlated with rs17632542-N1 and rs17632542-N2), based on all 600 null pairs in a cluster or 100 pairs under a selected MAF.

Among the 14 SNPs in the 7 causal pairs, 8 SNPs with a significant main effect formed a cluster (Table 2). For demonstration of the C-N pairs, we randomly selected one null SNP from the 6 MAF groups (MAF = 0.05, 0.1, 0.2, 0.3, 0.4, and 0.5). For each of these 8 SNPs, the results of 6 C-N pairs were shown in Supplementary Table S2. As we can see, all 6 null SNPs were not significantly associated with the outcome (p-main = 0.212–0.746). Under the same hub SNP, the p-pair of a C-N pair was reduced as the MAF of the null SNP was reduced. For a cluster with rs17632542 as a hub SNP, p-pair values were 0.455, 7.7 × 10^–14^, and 1.1 × 10^–15^ for a null SNP with a MAF of 0.5, 0.3, and 0.05, respectively. Furthermore, the results of the 600 null SNPs and these 8 hub SNPs were summarized in Table 2. For the clusters with a hub SNP with a p-main < 2.7 × 10^–7^, such as rs17632542, rs2569735, rs1058205, and rs4802755, the FPR range was 39.8%-79.5% for 1pRule and 15.0–38.7% for 3pRule. For the clusters with a hub SNP with a p-main > 2.7 × 10^–7^, the FPR range was 0–7.8%, the same for 1pRule and 3pRule. Consistent with Part 1, the hybrid study results (Part 2) confirm that 3pRule resulted in a noticeably lower FPR than 1pRule. The reduction in FPR by 3pRule compared with 1pRule was − 77% (from 66.5 to 15%) for the cluster of rs17632542, − 76% (from 79.5 to 19.2%) for the cluster of rs2569735, − 54% for the cluster of rs1058205 and − 20% for the cluster of rs4802755. These results demonstrated that 3pRule could effectively reduce FPR, especially for the top pairs. In summary, the magnitude of FPRs depends on the significance of this cut-point of p-pair. The FPRs for the C–N pairs tended to be high when the hub SNP had a small p-main, especially its p-main less than the criterion defining the significance of the SNP–SNP interactions (p-pair < 2.7 × 10^–7^). To identify the causes of the cluster effects for SNP–SNP interactions, we first tested the LD status between the hub SNP and significant null SNPs. Among the 4 large clusters with a *KLK3* SNP as a hub (rs17632542, rs2569735, rs1058205, and rs4802755), there are 90, 115, 111, and 232 significant null pairs in these 4 clusters. The LD r^2^ between each of these null SNPs and its corresponding hub SNP was close to 0 (range = 0–0.0004). For these 4 *KLK3* clusters, the pairwise LD r^2^ among the null SNPs in the same cluster were also close to 0 (range = 0–0.001, Suppl. Table S4). Thus, we can conclude that LD among the involved SNPs is not the reason for the cluster effect of SNP–SNP interactions. Next, we evaluated whether the significant null pairs in a cluster were highly correlated with the causal pair in the same cluster (such as C1–N1 and C1–N2 correlated with C1–C2). The results showed that null peripheral SNPs with a small MAF tended to be highly correlated with the causal pair to cause false positivity. As shown in Table 2, the null SNP with a smaller MAF tended to have a higher FPR than those with a larger MAF. For example, FPR values for the rs4802755 cluster decreased from 80 to 6% as the MAF of null SNPs increased from 5 to 50%. The mean correlations between rs4802755-rs4473378 and significant null pairs involved with the hub SNP of rs4802755 demonstrated a decreasing trend (r = 0.88 to 0.64) as MAF of null SNPs went up from 5 to 50%. Similar trends can be observed for other clusters of rs17632542, rs2569735, and rs1058205. Finally, we also tested correlations among 7 *KLK3* SNPs in Table 1. The pairwise LD r^2^ values among the 7 *KLK3* SNPs tested in Part 2 were not in a strong LD (all r^2^ < 0.8, n a range of 0.03–0.77). All r^2^ values were less than 0.6 except rs2569735 and rs1058205 (r^2^ = 0.77).

For evaluating the performance of the bootstrap + 3pRule approach, the related TPR results are summarized in Table 3. The TPR results corresponding to all the causal pairs in Table 3 revealed that the TPR values under 1pRule and 3pRule are very similar. In Table 3, all 7 causal pairs had > 75% TPR based on the 1pRule and 3pRule approaches. The TPR corresponding to the causal pair of rs17632542–rs4783709 was observed at 95% for 1pRule and 91.8% for 3pRule based on the 500 bootstrap runs. For these 7 causal pairs, these two methods (3pRule and 1pRule) of defining statistical significance for SNP–SNP interaction only varied by 0.2–3.2%. Thus, 3pRule with a more stringent criterion had a similar performance in terms of TPR compared to 1pRule for the causal pairs.

**Table 3:** True positive rates (TPR) by causal SNP pairs based on bootstrap results.

Causal pair	SNP1 p-value (p-main)^1^	SNP2 p-value (p-main)^1^	SNP–SNP interaction p-value (p-pair)^1^	Bootstrap^2^	
				1pRule TPR%^3^	3pRule TPR%^3^
rs17632542–rs4783709	**2.2 × 10**^**–15**^	0.027	**5.7 × 10**^**–18**^	95.0	91.8
rs2569735–rs7613553	**5.5 × 10**^**–9**^	0.551	**4.4 × 10**^**–13**^	98.2	98.0
rs1058205–rs2274545	**9.5 × 10**^**–8**^	0.065	**2.0 × 10**^**–9**^	83.6	81.0
rs4802755–rs4473378	**1.8 × 10**^**–7**^	0.728	**2.3 × 10**^**–9**^	86.6	86.0
rs174776–rs1250240	**7.9 × 10**^**–7**^	0.279	**2.4 × 10**^**–9**^	94.0	93.8
rs2271095–rs7446	**2.0 × 10**^**–6**^	**2.0 × 10**^**–5**^	**1.7 × 10**^**–12**^	95.4	94.4
rs266876–rs9521694	**3.2 × 10**^**–6**^	0.001	**3.4 × 10**^**–9**^	77.0	75.6

^1^Bold for significant results based on the Bonferroni criteria. p-main < 8.1 × 10^–5^ (= 0.05/614), and p-pair < 2.7 × 10^–7^ (= 0.05/^614^C_2_).

^2^Based on 500 bootstrap datasets with a sample size of 20,000 and 614 SNPs (7 causal pairs + 600 null SNPs).

^3^Significance rules: 1pRule: p-pair < 2.7 × 10^–7^; 3pRule: p-pair < 2.7 × 10^–7^, p-pair < p-main for SNP1, and p-pair < p-main for SNP2.

The FPR results of the bootstrap + 3pRule approach are shown in Table 4. Although FPR looked small (0.81% for 1pRule and 0.35% for 3pRule) in the original dataset, there were still many false-positive pairs due to a large number of pairwise interaction tests (188,191 pairs): 1538 significant pairs using 1pRule and 672 pairs using 3pRule. This showed that 3pRule could effectively reduce 56% of false-positive findings compared with 1pRule. Moreover, the bootstrap method can dramatically reduce the number of false-positive pairs. Using the bootstrap method under 3pRule, the number of significant pairs was only 86, 48, and 31 when using ≥ 75%, ≥ 80%, and ≥ 85% bootstrap criteria, respectively. By applying the criterion of ≥ 75% bootstrap runs and 3pRule for selecting significant pairs, this approach maintained 100% TPR, but its false-positive findings can be reduced by 95% from 1531 pairs identified using the conventional 1pRule without the bootstrap validation to 79 pairs (overall FPR = 0.82–0.04%). If using a more stringent criterion of ≥ 90% bootstrap datasets and 3pRule, false-positive findings out of the 1531 significant null pairs can be reduced further to 2% (= 26/1531), but TPR is reduced to 71.4% (5 out of the 7 pairs).

**Table 4:** Evaluation of the 3pRule + bootstrap approach for detecting SNP–SNP interactions based on a dataset with 614 SNPs.

Method^1^	Criterion	TPR^2^ (%)	FPR^2^ (%)	Total	No. significant pairs^1^				
					C–C (causal pair)	Total null pair	C–N (% null pairs)	C–C-other	N–N
1pRule	Original	100	0.82	1538	7	1531	1482 (96.8)	49	0
	≥ 75% runs^3^	100	0.24	468	7	461	439 (95.2)	22	0
	≥ 80% runs^3^	85.7	0.18	346	6	340	325 (95.6)	15	0
	≥ 90% runs^3^	71.4	0.14	262	5	257	245 (95.3)	12	0
3pRule	Original	100	0.35	672	7	665	624 (93.8)	41	0
	≥ 75% runs^3^	100	0.04	86	7	79	71 (89.9)	8	0
	≥ 80% runs^3^	85.7	0.02	48	6	42	37 (88.1)	5	0
	≥ 90% runs^3^	71.4	0.01	31	5	26	23 (88.5)	3	0

^1^Significance rules: 1pRule: p-pair < 2.7 × 10^–7^; 3pRule: p-pair < 2.7 × 10^–7^ and p-pair < p-main for SNP1, and p-pair < p-main for SNP2; “C”: a SNP in a causal pair; “N”: a null SNP.

^2^TPR (true positive rate): proportion of significance out of 7 causal pairs; FPR (false positive rate): proportion of significance null pairs out of all 188,184 null pairs.

^3^Based on 500 bootstrap runs.

For N–N pairs, the mean and median of p-main values for the 600 null SNPs were 0.33 and 0.29, respectively. For demonstration, we randomly selected one null SNP from the 6 MAF groups. The p-main and p-pair values of the 15 N–N pairs based on the selected 6 null SNPs were shown in Supplementary Table S3. The p-pair values for the pairwise interactions of these 6 null pairs (6.0 × 10^–3^ to 0.928) were insignificant. For the 179,700 N–N pairs, FPRs were 0% using both 1pRule and 3pRule with the original and bootstrap datasets (Table 4), and the mean and median p-pair values were 0.13 and 0.07, respectively, with an interquartile range of 0.028–0.147. The significance levels of the 7 C–C pairs and the selected null pairs are shown in Supplementary Fig. S2. As shown in Supplementary Fig. S2a, most of the N–N pairs were less significant than the C-N pairs. As shown in Supplementary Fig. S2b, for the distribution of the 1,797,700 N–N pairs’ significance levels, most of them (99.94%) had a p-pair ≥ 1 × 10^–4^. This result demonstrated that 1 × 10^–4^ can be used as the cut-point to select promising SNP–SNP interaction pairs. This is also why we used p-pair < 1 × 10^–4^ in Part 1. Because of the insignificance of N–N pairs, the exclusion of N–N pairs for SNP–SNP interaction detection can be used as a strategy of variable reduction.

## Discussion

This study addresses several important questions of SNP–SNP interaction detection, including reasons for FPR, methods for reducing FPR, and dimensional reduction. The cluster effects of significant SNP–SNP interaction pairs do not result from high LD between SNPs but are caused by high correlations between the causal pair(s) and null pairs (C-N pairs) in the same cluster. For factors associated with high FPR_cluster_, features of both the hub SNP and other peripheral SNPs matter. The hub SNP with a more significant main effect and a large MAF tends to interact with its peripheral SNPs to cause false positivity. In addition, peripheral null SNPs with a small MAF are likely to cause false positivity of SNP–SNP interactions. In this study, some SNPs (rs17632542, rs2569735, rs1058205, and rs4802755 in *KLK3* gene) are GWAS-identified SNPs associated with prostate cancer risk or aggressiveness^27^, so they have very significant main effects. Many studies performed SNP–SNP interaction analyses based on GWAS-identified SNPs or SNPs with significant main effects. When using powerful statistical approaches, searching SNP–SNP interactions considering GWAS-identified SNPs is effective because more significant SNP–SNP interaction pairs can be identified, but this approach also tends to have high false-positivity. Our findings can provide researchers a valuable insight into understanding false positivity in SNP–SNP interaction analyses.

False positivity is a well-known issue for high data dimensional analyses^28^. This high FPR issue worsens for studies of SNP–SNP interaction analyses because the number of interaction tests increases dramatically as the number of SNPs increases. In this study, there are 188,191 pairs for only 614 SNPs. The number of pairwise SNP–SNP interaction pairs increases to ~ 500,000 for 1000 SNPs and 12 million for 5000 SNPs. Thus, this extremely high-dimensional data issue makes the searching task of SNP–SNP interactions like finding a needle in a haystack. Our findings indicate that many null pairs were highly correlated with causal pairs, so this high correlation issue among the identified SNP-interaction pairs makes variable selection challenging. The bootstrap resampling method accounts for sampling variation and is useful for variable selection and internal validation. In bootstrapping, variables strongly associated with the outcome tend to be selected more often than variables with null or a weak effect^26,29^. Our results demonstrate that the bootstrap + 3pRule approach can effectively increase detection accuracy in identified SNP–SNP interactions. In addition to SNP–SNP interactions, other statistical approaches focus on reducing data dimension for evaluating epistasis. Some studies combined multiple SNPs into groups and then tested group–group interactions to evaluate epistasis^30,31^. The grouping methods of multiple SNPs were based on similar biological functions (such as pathways)^30^ or similarity using statistical methods (such as factors using factor analysis)^31^. However, these group–group interactions for testing epistasis lose valuable SNP-level information.

In addition, the low FPR for the N–N pairs provides valuable insights about dimensional reduction for detecting SNP–SNP interactions. Our study findings showed that two SNPs without a significant main effect tend to have no or weak interaction. This important feature can be applied to real data applications. We can test the significance of the main effects of SNP associated with an outcome. The variable reduction can be made by excluding the pairs composed of two SNPs with a weak or no main effect, such as N–N pairs in this study. This strategy can effectively reduce the number of interaction tests and ease the computation burden for testing SNP–SNP interactions.

In real data applications, the hub SNPs in the SNP-interaction clusters can be identified, but it is not easy to distinguish which pairs are true-positive or false-positive pairs. It is commonly known that a stringent significance criterion can reduce FPR, but statistical power (TPR) will also be reduced. Thus, it is essential to find an effective approach to address this issue. This study demonstrated that the bootstrap + 3pRule approach can reduce FPR and maintain high statistical power. Although the SNP–SNP interaction analyses in this study were based on SIPI, the cluster effects have been shown across studies with various traits and different statistical methods, such as non-parametric methods (Gini, absolute probability difference, and entropy)^13^, multipopulation harmony search, an artificial intelligence approach^10,15–17^, and chi-square test based on 8 interaction patterns^11^. Thus, our findings of cluster effect’s features and the bootstrap + 3pRule approach can be applied to other similar studies.

The strength of this study is the application of a solid study design with 2 parts (simulation analyses and hybrid analyses). Therefore, we can thoroughly evaluate the complicated cluster effects of SNP–SNP interactions. Our findings are closer to reality and more reliable because the causal pairs in this study are based on real data. This study also demonstrates that SIPI combined with the bootstrap + 3pRule approach is a powerful method for detecting SNP–SNP interactions. The limitation of this study is that it is primarily based on SIPI analyses. However, based on the literature review, we anticipate that other advanced statistical methods for detecting SNP–SNP inactions should benefit from our study findings in reducing false positivity. Further investigations will be needed to warrant our findings.

## Conclusions

Including SNP–SNP interaction pairs in polygenic risk scores is the key to driving substantial improvements in this domain. Even though the FPR, when applying the stringent Bonferroni criteria (1pRule), is not high in terms of the general rule of 5%, the number of false positive pairs is still large because of the large number of testing pairwise pairs. This study highlights the cluster effect and identifies the reasons for false positivity of SNP–SNP interactions. The bootstrap + 3pRule approach is suggested to increase the accuracy of SNP–SNP interaction detection.

### Supplementary Information

Supplementary Information.

## Data Availability

The simulation datasets used in this study are available from the corresponding author upon reasonable request. For data used for the real data application in this project, these data are available from the Prostate Cancer Association Group to Investigate Cancer Associated Alterations in the Genome Consortium (PRACTICAL Consortium, http://practical.icr.ac.uk/blog/?page_id=1242, email: practical@icr.ac.uk), but restrictions apply to the availability of these data.
