# Supplementary material for: Cluster effect for SNP–SNP interaction pairs for predicting complex traits
Source: Sci Rep. 2024 Aug 12;14:18677. doi: 10.1038/s41598-024-66311-7 (PMC11319716; doi:10.1038/s41598-024-66311-7)
Supplement: Supplementary file 1 — Supplementary Information. [file 41598_2024_66311_MOESM1_ESM.pdf]

## Supplementary Method Section

<u>Simulation process for the 12 “causal” pairs in Part 1</u>

1. **Templates of 4 significant SNP-SNP interaction pairs:** Use a template of the 4 SNP-SNP interaction pairs associated with a binary outcome based on our published paper to generate 12 causal pairs.

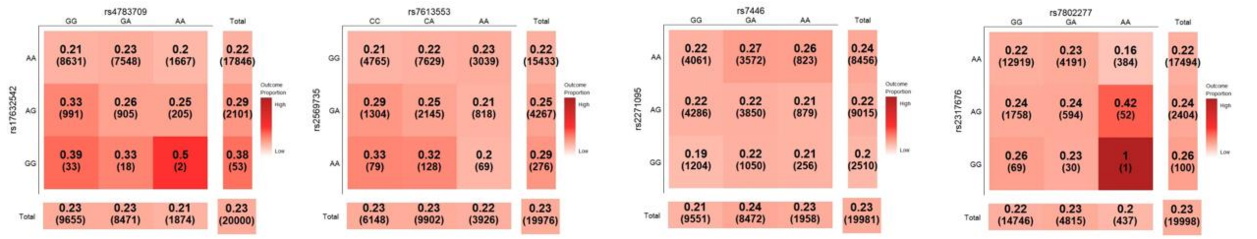


**Footnote:** Selected SNP-SNP interactions associated with prostate cancer aggressiveness. The 1st number in the cell is the prevalence of prostate cancer aggressiveness, and the 2nd value inside the paraphrasis is the sample size in the genotype combination. The darker the color indicates the higher risk of prostate cancer aggressiveness.

2. **Assign each subject a genotype combination of an SNP pair:** The samples per each of the 9 genotype combinations were generated using a multinomial distribution based on the sample distribution from the 3*3 table for a real pair.

3. **Generate the outcome for each subject:** For each SNP pair, there were 9 genotype combinations (such as AA-GG, AA-GA, and AA-AA). Using a logistic model with an SNP pair with 9 genotype combinations, we obtained 8 model coefficients, excluding an intercept based on real data. Then, we calculated the predicted probability of the outcome (such as Y=1 as a disease) based on this logistic model for the subjects in each genotype combination. Next, the outcome status for each subject was decided using a binomial distribution based on the predicted probability for each genotype.

4. **Test 3 various significance levels for each interaction pattern:** For each of the 4 interaction patterns, 3 various significance levels (low significance (L), medium significance (M), and high significance (H)) were tested. For generating pairs with various significance levels under a similar interaction pattern, we generated the predicted probabilities of the outcome based on the logistic models with and without adding a random variable from a normal distribution with a mean of 0 and standard deviation of 1 and 2 [N(0, 1) and N(0, 2)].

5. Other settings: Based on Steps 1-3, 12 causal pairs were generated. Each was tested under 3 sample sizes (n=5,000, 10,000, and 20,000).

Supplementary Table S1. Simulation setting of the 4 sets of causal pairs with a total of 12 causal pairs under 3 sample sizes in Part 1

| Set | SNP1 Min<Maj (MAF)[2] | SNP2 Min<Maj (MAF)[2] | Pair[1] | Sample size | SNP-SNP p-pair[3] | SNP1 p-Main[3] | SNP2 p-Main[3] |
|---|---|---|---|---|---|---|---|
| C1-C2 | G<A (0.055) | A<G (0.306) | $C1_H$-$C2_H$ | 20,000 | 4.5X10$^{-18}$ | 1.2X10$^{-15}$ | 0.022 |
| | | | $C1_M$-$C2_M$ | | 9.1X10$^{-14}$ | 6.1X10$^{-12}$ | 0.036 |
| | | | $C1_L$-$C2_L$ | | 1.6X10$^{-08}$ | 2.2X10$^{-7}$ | 0.090 |
| | | | | | | | |
| | | | $C1_H$-$C2_H$ | 10,000 | 7.5X10$^{-10}$ | 1.4X10$^{-8}$ | 0.093 |
| | | | $C1_M$-$C2_M$ | | 9.6X10$^{-8}$ | 8.4X10$^{-7}$ | 0.122 |
| | | | $C1_L$-$C2_L$ | | 3.8X10$^{-5}$ | 1.9X10$^{-4}$ | 0.179 |
| | | | | | | | |
| | | | $C1_H$-$C2_H$ | 5,000 | 9.0X10$^{-6}$ | 6.6X10$^{-5}$ | 0.159 |
| | | | $C1_M$-$C2_M$ | | 1.5X10$^{-4}$ | 6.1X10$^{-4}$ | 0.189 |
| | | | $C1_L$-$C2_L$ | | 2.7X10$^{-3}$ | 8.2X10$^{-3}$ | 0.227 |
| | | | | | | | |
| C3-C4 | A<G (0.121) | A<C (0.444) | $C3_H$-$C4_H$ | 20,000 | 3.9X10$^{-13}$ | 5.3X10$^{-9}$ | 0.244 |
| | | | $C3_M$-$C4_M$ | | 2.1X10$^{-10}$ | 2.9X10$^{-7}$ | 0.259 |
| | | | $C3_L$-$C4_L$ | | 1.1X10$^{-6}$ | 9.3X10$^{-5}$ | 0.277 |
| | | | | | | | |
| | | | $C3_H$-$C4_H$ | 10,000 | 2.1X10$^{-7}$ | 3.5X10$^{-5}$ | 0.277 |
| | | | $C3_M$-$C4_M$ | | 5.0X10$^{-6}$ | 2.5X10$^{-4}$ | 0.261 |
| | | | $C3_L$-$C4_L$ | | 4.1X10$^{-4}$ | 4.4X10$^{-3}$ | 0.297 |
| | | | | | | | |
| | | | $C3_H$-$C4_H$ | 5,000 | 1.8X10$^{-4}$ | 3.4X10$^{-3}$ | 0.294 |
| | | | $C3_M$-$C4_M$ | | 6.8X10$^{-4}$ | 7.0X10$^{-3}$ | 0.276 |
| | | | $C3_L$-$C4_L$ | | 5.7X10$^{-3}$ | 3.4X10$^{-2}$ | 0.290 |
| | | | | | | | |
| C5-C6 | G<A (0.351) | A<G (0.310) | $C5_H$-$C6_H$ | 20,000 | 2.1X10$^{-12}$ | 9.2X10$^{-7}$ | 1.6X10$^{-5}$ |
| | | | $C5_M$-$C6_M$ | | 8.4X10$^{-10}$ | 1.4X10$^{-5}$ | 2.0X10$^{-4}$ |
| | | | $C5_L$-$C6_L$ | | 1.5X10$^{-6}$ | 9.0X10$^{-4}$ | 0.003 |
| | | | | | | | |
| | | | $C5_H$-$C6_H$ | 10,000 | 4.9X10$^{-7}$ | 3.5X10$^{-4}$ | 0.002 |
| | | | $C5_M$-$C6_M$ | | 8.8X10$^{-6}$ | 0.001 | 0.007 |
| | | | $C5_L$-$C6_L$ | | 3.7X10$^{-4}$ | 0.012 | 0.039 |
| | | | | | | | |
| | | | $C5_H$-$C6_H$ | 5,000 | 3.0X10$^{-4}$ | 0.011 | 0.033 |
| | | | $C5_M$-$C6_M$ | | 9.0X10$^{-4}$ | 0.018 | 0.050 |
| | | | $C5_L$-$C6_L$ | | 6.2X10$^{-3}$ | 0.051 | 0.132 |
| | | | | | | | |
| C7-C8 | G<A (0.065) | A<G (0.142) | $C7_H$-$C8_H$ | 20,000 | 6.7X10$^{-5}$ | 0.019 | 0.070 |
| | | | $C7_M$-$C8_M$ | | 5.6X10$^{-4}$ | 0.042 | 0.071 |
| | | | $C7_L$-$C8_L$ | | 4.5X10$^{-3}$ | 0.106 | 0.120 |
| | | | | | | | |
| | | | $C7_H$-$C8_H$ | 10,000 | 3.1X10$^{-3}$ | 0.077 | 0.134 |
| | | | $C7_M$-$C8_M$ | | 6.0X10$^{-3}$ | 0.123 | 0.163 |
| | | | $C7_L$-$C8_L$ | | 0.016 | 0.163 | 0.183 |
| | | | | | | | |
| | | | $C7_H$-$C8_H$ | 5,000 | 0.017 | 0.163 | 0.207 |
| | | | $C7_M$-$C8_M$ | | 0.024 | 0.176 | 0.195 |
| | | | $C7_L$-$C8_L$ | | 0.046 | 0.240 | 0.241 |

[1] Significance level of an interaction pair: H (high significance), M (medium significance), L (low significance)

[2] Min: minor allele, Maj: major allele, MAF: minor allele frequency

[3] p-main median of the p-value of SNP main effect; p-pair: median p-value of SNP-SNP interaction pair based on 1000 simulation runs.

Supplementary Table S2. Summary of the selected causal-null (C-N) SNP-SNP interaction pairs associated with a binary outcome for 8 real SNPs

| SNP pair SNP1-SNP2 | SNP1 Min<Maj (MAF)[1] | SNP2 Min<Maj (MAF)[1] | SNP1 p-value (p-main)[2] | SNP2 p-value (p-main)[2] | SNP-SNP Interaction p-value (p-pair)[2] |
|---|---|---|---|---|---|
| rs17632542-N0 | G<A (0.06) | A<G (0.05) | **$2.2 \times 10^{-15}$** | 0.365 | **$1.1 \times 10^{-15}$** |
| rs17632542-N1 | G<A (0.06) | A<G (0.10) | **$2.2 \times 10^{-15}$** | 0.305 | **$1.3 \times 10^{-15}$** |
| rs17632542-N2 | G<A (0.06) | A<G (0.20) | **$2.2 \times 10^{-15}$** | 0.746 | $4.3 \times 10^{-14}$ |
| rs17632542-N3 | G<A (0.06) | A<G (0.30) | **$2.2 \times 10^{-15}$** | 0.615 | $7.7 \times 10^{-14}$ |
| rs17632542-N4 | G<A (0.06) | A<G (0.40) | **$2.2 \times 10^{-15}$** | 0.690 | 0.354 |
| rs17632542-N5 | G<A (0.06) | A<G (0.50) | **$2.2 \times 10^{-15}$** | 0.212 | 0.455 |
| | | | | | |
| rs2569735-N0 | A<G (0.12) | A<G (0.05) | **$5.5 \times 10^{-9}$** | 0.365 | **$3.4 \times 10^{-9}$** |
| rs2569735-N1 | A<G (0.12) | A<G (0.10) | **$5.5 \times 10^{-9}$** | 0.305 | $1.0 \times 10^{-8}$ |
| rs2569735-N2 | A<G (0.12) | A<G (0.20) | **$5.5 \times 10^{-9}$** | 0.746 | $3.0 \times 10^{-7}$ |
| rs2569735-N3 | A<G (0.12) | A<G (0.30) | **$5.5 \times 10^{-9}$** | 0.615 | $1.6 \times 10^{-7}$ |
| rs2569735-N4 | A<G (0.12) | A<G (0.40) | **$5.5 \times 10^{-9}$** | 0.690 | $1.5 \times 10^{-7}$ |
| rs2569735-N5 | A<G (0.12) | A<G (0.50) | **$5.5 \times 10^{-9}$** | 0.212 | $2.6 \times 10^{-8}$ |
| | | | | | |
| rs1058205-N0 | G<A (0.15) | A<G (0.05) | **$9.5 \times 10^{-8}$** | 0.365 | **$8.1 \times 10^{-8}$** |
| rs1058205-N1 | G<A (0.15) | A<G (0.10) | **$9.5 \times 10^{-8}$** | 0.305 | $3.3 \times 10^{-7}$ |
| rs1058205-N2 | G<A (0.15) | A<G (0.20) | **$9.5 \times 10^{-8}$** | 0.746 | $1.6 \times 10^{-6}$ |
| rs1058205-N3 | G<A (0.15) | A<G (0.30) | **$9.5 \times 10^{-8}$** | 0.615 | $2.6 \times 10^{-6}$ |
| rs1058205-N4 | G<A (0.15) | A<G (0.40) | **$9.5 \times 10^{-8}$** | 0.690 | $2.4 \times 10^{-6}$ |
| rs1058205-N5 | G<A (0.15) | A<G (0.50) | **$9.5 \times 10^{-8}$** | 0.212 | $1.1 \times 10^{-6}$ |
| | | | | | |
| rs4802755-N0 | A<G (0.46) | A<G (0.05) | **$1.8 \times 10^{-7}$** | 0.365 | **$1.2 \times 10^{-7}$** |
| rs4802755-N1 | A<G (0.46) | A<G (0.10) | **$1.8 \times 10^{-7}$** | 0.305 | **$1.6 \times 10^{-7}$** |
| rs4802755-N2 | A<G (0.46) | A<G (0.20) | **$1.8 \times 10^{-7}$** | 0.746 | $1.4 \times 10^{-2}$ |
| rs4802755-N3 | A<G (0.46) | A<G (0.30) | **$1.8 \times 10^{-7}$** | 0.615 | $6.0 \times 10^{-7}$ |
| rs4802755-N4 | A<G (0.46) | A<G (0.40) | **$1.8 \times 10^{-7}$** | 0.690 | $3.7 \times 10^{-7}$ |
| rs4802755-N5 | A<G (0.46) | A<G (0.50) | **$1.8 \times 10^{-7}$** | 0.212 | $2.3 \times 10^{-6}$ |
| | | | | | |
| rs174776-N0 | A<G (0.11) | A<G (0.05) | **$7.9 \times 10^{-7}$** | 0.365 | $1.8 \times 10^{-6}$ |
| rs174776-N1 | A<G (0.11) | A<G (0.10) | **$7.9 \times 10^{-7}$** | 0.305 | $3.6 \times 10^{-6}$ |
| rs174776-N2 | A<G (0.11) | A<G (0.20) | **$7.9 \times 10^{-7}$** | 0.746 | $1.1 \times 10^{-6}$ |
| rs174776-N3 | A<G (0.11) | A<G (0.30) | **$7.9 \times 10^{-7}$** | 0.615 | $3.2 \times 10^{-6}$ |
| rs174776-N4 | A<G (0.11) | A<G (0.40) | **$7.9 \times 10^{-7}$** | 0.690 | $3.1 \times 10^{-6}$ |
| rs174776-N5 | A<G (0.11) | A<G (0.50) | **$7.9 \times 10^{-7}$** | 0.212 | $1.9 \times 10^{-5}$ |
| | | | | | |
| rs2271095-N0 | G<A (0.35) | A<G (0.05) | **$2.0 \times 10^{-6}$** | 0.365 | $3.6 \times 10^{-6}$ |
| rs2271095-N1 | G<A (0.35) | A<G (0.10) | **$2.0 \times 10^{-6}$** | 0.305 | $2.5 \times 10^{-6}$ |
| rs2271095-N2 | G<A (0.35) | A<G (0.20) | **$2.0 \times 10^{-6}$** | 0.746 | $3.4 \times 10^{-5}$ |
| rs2271095-N3 | G<A (0.35) | A<G (0.30) | **$2.0 \times 10^{-6}$** | 0.615 | $5.9 \times 10^{-6}$ |
| rs2271095-N4 | G<A (0.35) | A<G (0.40) | **$2.0 \times 10^{-6}$** | 0.690 | $4.1 \times 10^{-5}$ |
| rs2271095-N5 | G<A (0.35) | A<G (0.50) | **$2.0 \times 10^{-6}$** | 0.212 | $2.8 \times 10^{-5}$ |
| | | | | | |
| rs7446-N0 | A<G (0.31) | A<G (0.05) | **$2.0 \times 10^{-5}$** | 0.365 | $2.9 \times 10^{-5}$ |
| rs7446-N1 | A<G (0.31) | A<G (0.10) | **$2.0 \times 10^{-5}$** | 0.305 | $1.1 \times 10^{-5}$ |
| rs7446-N2 | A<G (0.31) | A<G (0.20) | **$2.0 \times 10^{-5}$** | 0.746 | $2.4 \times 10^{-5}$ |
| rs7446-N3 | A<G (0.31) | A<G (0.30) | **$2.0 \times 10^{-5}$** | 0.615 | $1.2 \times 10^{-4}$ |
| rs7446-N4 | A<G (0.31) | A<G (0.40) | **$2.0 \times 10^{-5}$** | 0.690 | $7.0 \times 10^{-4}$ |
| rs7446-N5 | A<G (0.31) | A<G (0.50) | **$2.0 \times 10^{-5}$** | 0.212 | $1.0 \times 10^{-4}$ |
| | | | | | |
| rs266876-N0 | G<A (0.24) | A<G (0.05) | **$3.2 \times 10^{-6}$** | 0.365 | $2.2 \times 10^{-6}$ |
| rs266876-N1 | G<A (0.24) | A<G (0.10) | **$3.2 \times 10^{-6}$** | 0.305 | $1.4 \times 10^{-5}$ |
| rs266876-N2 | G<A (0.24) | A<G (0.20) | **$3.2 \times 10^{-6}$** | 0.746 | $1.4 \times 10^{-4}$ |
| rs266876-N3 | G<A (0.24) | A<G (0.30) | **$3.2 \times 10^{-6}$** | 0.615 | $2.2 \times 10^{-5}$ |
| rs266876-N4 | G<A (0.24) | A<G (0.40) | **$3.2 \times 10^{-6}$** | 0.690 | $4.2 \times 10^{-4}$ |
| rs266876-N5 | G<A (0.24) | A<G (0.50) | **$3.2 \times 10^{-6}$** | 0.212 | $3.5 \times 10^{-5}$ |

[1] Min: minor allele, Maj: major allele, MAF: minor allele frequency

[2] Bold for significant results based on the Bonferroni criteria. p-main< $8.1 \times 10^{-5}$ (=0.05/614), and p-pair< $2.7 \times 10^{-7}$ (= $0.05/{}^{614}C_2$)

Supplementary Table S3. Summary of the selected null-null (N-N) SNP-SNP interaction pairs associated with a binary outcome

| SNP pair SNP1-SNP2 | SNP1 Min<Maj (MAF)[1] | SNP2 Min<Maj (MAF)[1] | SNP1 p-value (p-main) | SNP2 p-value (p-main) | SNP-SNP Interaction p-value (p-pair) |
|---|---|---|---|---|---|
| N0-N1 | A<G (0.05) | A<G (0.10) | 0.365 | 0.305 | 0.180 |
| N0-N2 | A<G (0.05) | A<G (0.20) | 0.365 | 0.746 | 0.928 |
| N0-N3 | A<G (0.05) | A<G (0.30) | 0.365 | 0.615 | 0.168 |
| N0-N4 | A<G (0.05) | A<G (0.40) | 0.365 | 0.690 | 0.205 |
| N0-N5 | A<G (0.05) | A<G (0.50) | 0.365 | 0.212 | $6.0 \times 10^{-3}$ |
| | | | | | |
| N1-N2 | A<G (0.10) | A<G (0.20) | 0.305 | 0.746 | 0.092 |
| N1-N3 | A<G (0.10) | A<G (0.30) | 0.305 | 0.615 | 0.014 |
| N1-N4 | A<G (0.10) | A<G (0.40) | 0.305 | 0.690 | 0.013 |
| N1-N5 | A<G (0.10) | A<G (0.50) | 0.305 | 0.212 | $9.0 \times 10^{-3}$ |
| | | | | | |
| N2-N3 | A<G (0.20) | A<G (0.30) | 0.746 | 0.615 | 0.041 |
| N2-N4 | A<G (0.20) | A<G (0.40) | 0.746 | 0.690 | 0.643 |
| N2-N5 | A<G (0.20) | A<G (0.50) | 0.746 | 0.212 | 0.161 |
| | | | | | |
| N3-N4 | A<G (0.30) | A<G (0.40) | 0.615 | 0.690 | 0.330 |
| N3-N5 | A<G (0.30) | A<G (0.50) | 0.615 | 0.212 | 0.294 |
| | | | | | |
| N4-N5 | A<G (0.40) | A<G (0.50) | 0.690 | 0.212 | 0.237 |

[1] Min: minor allele, Maj: major allele, MAF: minor allele frequency

Supplementary Table S4. Linkage disequilibrium (LD) among the null SNPs in a cluster

| Cluster (bold: hub SNP) | No. of pairwise LD tests (No. Null SNPs) | Average LD | Min LD | Max LD |
|---|---|---|---|---|
| **rs17632542**-rs4783709 | 4005 (90) | 0.00005 | 0 | 0.0009 |
| **rs2569735**-rs7613553 | 6555 (115) | 0.00005 | 0 | 0.0007 |
| **rs1058205**-rs2274545 | 6105 (111) | 0.00005 | 0 | 0.0006 |
| **rs4802755**-rs4473378 | 26796 (232) | 0.00005 | 0 | 0.001 |

Supplementary Figure S1. True identification rates ($TIR_{s1k}$) for 4 sets of causal SNP pairs based on 1000 runs

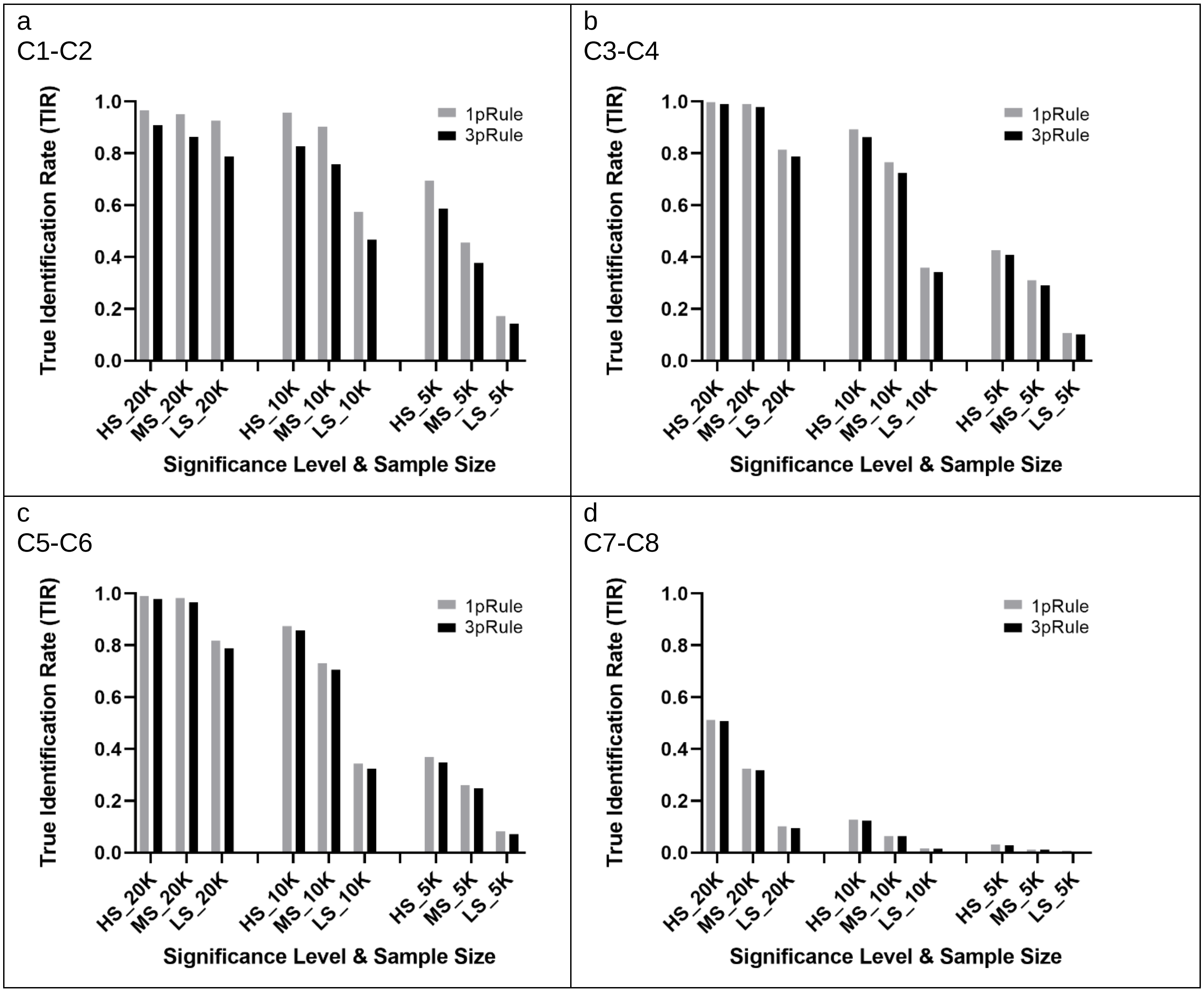


Note: Each set had 3 clusters with a causal pair with various significance levels, such as HS, MS, and LS, for a high, medium, and low significance level, respectively. Sample size: 20K (n=20000), 10K (n=10000), and 5K (n=5000). Significance rules: 1pRule: p-pair < $2.7 \times 10^{-7}$ ; 3pRule: p-pair< $2.7 \times 10^{-7}$ and p-pair < p-main for SNP1, and p-pair< p-main for SNP2.

Supplementary Figure S2. Distribution of significance of SNP-SNP interactions by pair types

a

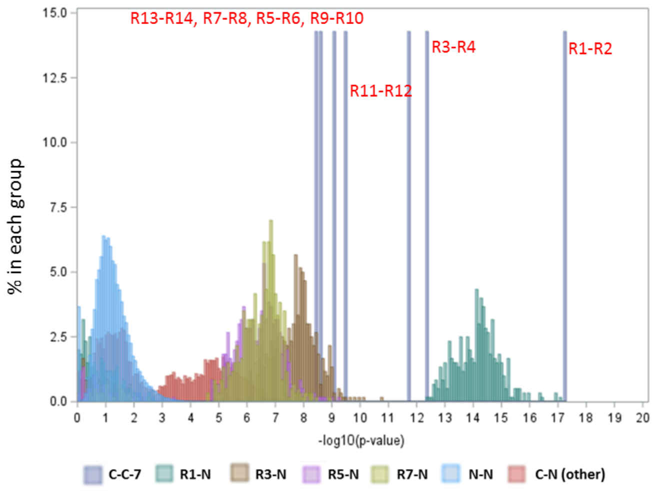


| Causal (C-C-7) pairs | SNP pair SNP1-SNP2 | SNP1 p-main | SNP2 p-main | p-pair |
|---|---|---|---|---|
| R1-R2 | rs17632542-rs4783709 | $2.2\times10^{-15}$ | 0.027 | $5.7\times10^{-18}$ |
| R3-R4 | rs2569735-rs7613553 | $5.5\times10^{-9}$ | 0.551 | $4.4\times10^{-13}$ |
| R5-R6 | rs1058205-rs2274545 | $9.5\times10^{-8}$ | 0.065 | $8.5\times10^{-10}$ |
| R7-R8 | rs4802755-rs4473378 | $1.8\times10^{-7}$ | 0.728 | $2.3\times10^{-9}$ |
| R9-R10 | rs174776-rs1250240 | $7.9\times10^{-7}$ | 0.279 | $3.5\times10^{-10}$ |
| R11-R12 | rs2271095-rs7446 | $2.0\times10^{-6}$ | $2.0\times10^{-5}$ | $1.7\times10^{-12}$ |
| R13-R14 | rs266876-rs9521694 | $3.2\times10^{-6}$ | 0.001 | $3.4\times10^{-9}$ |

b

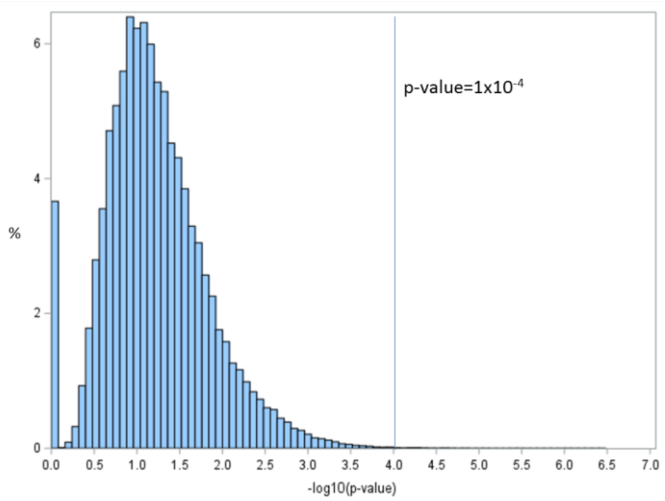


Note: a: 7 causal pairs and types of null pairs (C-N and N-N), and b: null pairs (N-N pairs). Note: ”C” represents a SNP from an observed causal pair; “N” represents a simulated null SNP; Results are based on 7 causal (C-C) pairs, 8400 causal-null (C-N) pairs, and 179700 null-null (N-N) pairs. The percentages were calculated based on the number of pairs in each group.

**The Prostate Cancer Association Group to Investigate Cancer Associated Alterations in the Genome (PRACTICAL) CONSORTIUM (Supplementary authors in addition to those named in the main author list)**

Information on the consortium can be found at http://practical.icr.ac.uk/

Additional members from the consortium are:

Artitaya Lophatananon[1], Teuvo L. J. Tammela[2,3], Anssi Auvinen[4], Alison M. Dunning[5], Srilakshmi Srinivasan[6], Judith A. Clements[7,6], Suzanne Chambers[8,9], Lisa Horvath[10,11], Wayne Tilley[12], Gail P. Risbridger[13,14], Achala Shenali Fernando Vitharanage[7,6], Jenny L. Donovan[15], Freddie C. Hamdy[16,17], Richard M. Martin[15,18,19], Stig E. Bojesen[20,21], Hein V. Stroomberg[22], Andreas Røder[22,23], Melissa C. Southey[24,25 ,26], Graham G. Giles[25,27,24], Roger L. Milne[25,27,24], Fredrick R. Schumacher[28,29], Loic Le Marchand[30], Xin Sheng[31], Tim J. Key[32], Elaine A. Ostrander[33], Bettina F. Drake[34], Robert Szulkin[35,36], Markus Aly[37,38,39], Dominika Wokołorczyk[40], Jan Lubinski[40], Thomas A. Sellers[41], Manuel Luedeke[42], Thomas Schnoeller[43], Shannon K. McDonnell[44], Daniel J. Schaid[44], Paula Paulo[45], Andreia Brandão[45], Craig C Teerlink[46,47], Xin Gao[48], Bernd Holleczek[49], Ben Schöttker[48], Chavdar Slavov[50], Vanio Mitev[51], Agnieszka Michael[52]

[1]Division of Population Health, Health Services Research and Primary Care, University of Manchester, Oxford Road, Manchester, M13 9PL, UK. [2]Department of Urology, Tampere University Hospital, FI-33521 Tampere, Finland. [3]Faculty of Medicine and Health Technology, Tampere University, FI-33100 Tampere, Finland. [4]Unit of Health Sciences, Faculty of Social Sciences, Tampere University, Tampere, Finland. [5]Centre for Cancer Genetic Epidemiology, Department of Oncology, University of Cambridge, Strangeways Laboratory, Worts Causeway, Cambridge, CB1 8RN, UK. [6]Translational Research Institute, Brisbane, Queensland 4102, Australia. [7]Australian Prostate Cancer Research Centre-Qld, Institute of Health and Biomedical Innovation and School of Biomedical Science, Queensland University of Technology, Brisbane, QLD 4059, Australia. [8]Faculty of Health Sciences, Australian Catholic University, Fitzroy, VIC 3065, Australia. [9]Cancer Council Queensland, Fortitude Valley, QLD 4006, Australia. [10]Chris O'Brien Lifehouse (COBLH), Camperdown, Sydney, NSW 2010, Australia. [11]Garvan Institute of Medical Research, Sydney NSW 2010, Australia.[12]Dame Roma Mitchell Cancer Research Laboratories, University of Adelaide, Adelaide, South Australia, Australia. [13]Department of Anatomy and Developmental Biology, Biomedicine Discovery Institute, Monash University, Melbourne, Victoria 3800, Australia. [14]Prostate Cancer Translational Research Program, Cancer Research Division, Peter MacCallum Cancer Centre, Melbourne, VIC 3000, Australia. [15]Population Health Sciences, Bristol Medical School, University of Bristol, BS8 2PS, UK. [16]Nuffield Department of Surgical Sciences, University of Oxford, Oxford, OX1 2JD, UK. [17]Faculty of Medical Science, University of Oxford, John Radcliffe Hospital, Oxford, UK. [18]National Institute for Health Research (NIHR) Biomedical Research Centre, University of Bristol, Bristol, BS8 1TH, UK. [19]Medical Research Council (MRC) Integrative Epidemiology Unit, University of Bristol, Bristol, BS8 2BN, UK. [20]Faculty of Health and Medical Sciences, University of Copenhagen, 2200 Copenhagen, Denmark. [21]Department of Clinical Biochemistry, Herlev and Gentofte Hospital, Copenhagen University Hospital, Herlev, 2200 Copenhagen, Denmark. [22]Copenhagen Prostate Cancer Center, Department of Urology, Copenhagen University Hospital - Rigshospitalet, Copenhagen, Denmark. [23]Department of Clinical Medicine, University of Copenhagen, Copenhagen, Denmark.[24]Precision Medicine, School of Clinical Sciences at Monash Health, Monash University, Clayton, Victoria 3168, Australia. [25]Cancer Epidemiology Division, Cancer Council Victoria, 200 Victoria Parade, East Melbourne, VIC, 3002, Australia. [26]Department of Clinical Pathology, The Melbourne Medical School, The University of Melbourne, Melbourne, VIC 3010, Australia. [27]Centre for Epidemiology and Biostatistics, Melbourne School of Population and Global Health, The University of Melbourne, Grattan

Street, Parkville, VIC 3010, Australia. [28]Department of Population and Quantitative Health Sciences, Case Western Reserve University, Cleveland, OH 44106-7219, USA. [29]Seidman Cancer Center, University Hospitals, Cleveland, OH 44106, USA. [30]Epidemiology Program, University of Hawaii Cancer Center, Honolulu, HI 96813, USA. [31]Center for Genetic Epidemiology, Department of Preventive Medicine, Keck School of Medicine, University of Southern California/Norris Comprehensive Cancer Center, Los Angeles, CA 90015, USA. [32]Cancer Epidemiology Unit, Nuffield Department of Population Health, University of Oxford, Oxford, OX3 7LF, UK. [33]National Human Genome Research Institute, National Institutes of Health, 50 South Drive, Rm. 5351, Bethesda, MD 20892, USA. [34]Washington University School of Medicine, 660 S. Euclid Avenue, Campus Box 8242, St. Louis, MO 63110, USA. [35]Department of Medical Epidemiology and Biostatistics, Karolinska Institute, Stockholm, Sweden. [36]SDS Life Science, Danderyd, Sweden. [37]Department of Medical Epidemiology and Biostatistics, Karolinska Institute, SE-171 77 Stockholm, Sweden. [38]Department of Molecular Medicine and Surgery, Karolinska Institutet, Karolinska University Hospital, Solna, 171 76 Stockholm, Sweden. [39]Department of Urology, Karolinska University Hospital, Solna, 171 76 Stockholm, Sweden. [40]International Hereditary Cancer Center, Department of Genetics and Pathology, Pomeranian Medical University, 70-115 Szczecin, Poland. [41]Department of Cancer Epidemiology, Moffitt Cancer Center, 12902 Magnolia Drive, Tampa, FL 33612, USA. [42]Genetikum, Wegenerstr. 15, D-89231 Neu-Ulm, Germany. [43]Department of Urology, University Hospital Ulm, Germany. [44]Division of Biomedical Statistics & Informatics, Mayo Clinic, Rochester, MN 55905, USA. [45]Cancer Genetics Group, IPO Porto Research Center (CI-IPOP) / RISE@CI-IPOP (Health Research Network), Portuguese Oncology Institute of Porto (IPO Porto) / Porto Comprehensive Cancer Center, Porto, Portugal. [46]Division of Epidemiology, Department of Internal Medicine, University of Utah School of Medicine, Salt Lake City, Utah 84132, USA. [47]George E. Wahlen Department of Veterans Affairs Medical Center, Salt Lake City, Utah 84148, USA. [48]Division of Clinical Epidemiology and Aging Research, German Cancer Research Center (DKFZ), Heidelberg, Germany. [49]Saarland Cancer Registry, 66119 Saarbrücken, Germany. [50]Department of Urology and Alexandrovska University Hospital, Medical University of Sofia, 1431 Sofia, Bulgaria. [51]Molecular Medicine Center, Department of Medical Chemistry and Biochemistry, Medical University of Sofia, Sofia, 2 Zdrave Str., 1431 Sofia, Bulgaria. [52]The University of Surrey, Guildford, Surrey, GU2 7XH, UK.

**Funding for the CRUK study and PRACTICAL consortium:**

This work was supported by the Canadian Institutes of Health Research, European Commission's Seventh Framework Programme grant agreement n° 223175 (HEALTH-F2-2009-223175), Cancer Research UK Grants C5047/A7357, C1287/A10118, C1287/A16563, C5047/A3354, C5047/A10692, C16913/A6135, and The National Institute of Health (NIH) Cancer Post-Cancer GWAS initiative grant: No. 1 U19 CA 148537-01 (the GAME-ON initiative).

**COGS acknowledgment:**

This study would not have been possible without the contributions of the following: Per Hall (COGS); Douglas F. Easton, Paul Pharoah, Kyriaki Michailidou, Manjeet K. Bolla, Qin Wang (BCAC), Andrew Berchuck (OCAC), Rosalind A. Eeles, Douglas F. Easton, Ali Amin Al Olama, Zsofia Kote-Jarai, Sara Benlloch (PRACTICAL), Georgia Chenevix-Trench, Antonis Antoniou, Lesley McGuffog, Fergus Couch and Ken Offit (CIMBA), Joe Dennis, Alison M. Dunning, Andrew Lee, and Ed Dicks, Craig Luccarini and the staff of the Centre for Genetic Epidemiology Laboratory, Javier Benitez, Anna Gonzalez-Neira and the staff of the CNIO genotyping unit, Jacques Simard and Daniel C. Tessier, Francois Bacot, Daniel Vincent, Sylvie LaBoissière and Frederic Robidoux and the staff of the McGill University and Génome Québec Innovation Centre, Stig E. Bojesen, Sune F. Nielsen, Borge G. Nordestgaard, and the staff of the

Copenhagen DNA laboratory, and Julie M. Cunningham, Sharon A. Windebank, Christopher A. Hilker, Jeffrey Meyer and the staff of Mayo Clinic Genotyping Core Facility.

Funding for the iCOGS infrastructure came from: the European Community's Seventh Framework Programme under grant agreement n° 223175 (HEALTH-F2-2009-223175) (COGS), Cancer Research UK (C1287/A10118, C1287/A 10710, C12292/A11174, C1281/A12014, C5047/A8384, C5047/A15007, C5047/A10692, C8197/A16565), the National Institutes of Health (CA128978) and Post-Cancer GWAS initiative (1U19 CA148537, 1U19 CA148065 and 1U19 CA148112 - the GAME-ON initiative), the Department of Defence (W81XWH-10-1-0341), the Canadian Institutes of Health Research (CIHR) for the CIHR Team in Familial Risks of Breast Cancer, Komen Foundation for the Cure, the Breast Cancer Research Foundation, and the Ovarian Cancer Research Fund.

**Additional funding and acknowledgments from studies in PRACTICAL:**

CAPS / STHM1
The Department of Medical Epidemiology and Biostatistics, Karolinska Institute, Stockholm, Sweden was supported by the Cancer Risk Prediction Center (CRisP; www.crispcenter.org), a Linneus Centre (Contract ID 70867902) financed by the Swedish Research Council, Swedish Research Council (grant no K2010-70X-20430-04-3), the Swedish Cancer Foundation (grant no 09-0677), the Hedlund Foundation, the Soederberg Foundation, the Enqvist Foundation, ALF funds from the Stockholm County Council. Stiftelsen Johanna Hagstrand och Sigfrid Linner's Minne, Karlsson's Fund for urological and surgical research. We thank and acknowledge all of the participants in the Stockholm-1 study. We thank Carin Cavalli-Bjoerkman and Ami Roennberg Karlsson for their dedicated work in the collection of data. Michael Broms is acknowledged for his skillful work with databases. KI Biobank is acknowledged for handling the samples and for DNA extraction. Hans Wallinder at Aleris Medilab and Sven Gustafsson at Karolinska University Laboratory are thanked for their good cooperation in providing historical laboratory results.

CPCS1 / CPCS2
Department of Clinical Biochemistry, Herlev and Gentofte Hospital, Copenhagen University Hospital, Herlev Ringvej 75, DK-2730 Herlev, Denmark. We thank participants and staff of the Copenhagen General Population Study for their important contributions.

EPIC
The coordination of EPIC was financially supported by the European Commission (DG-SANCO) and the International Agency for Research on Cancer. The national cohorts (that recruited male participants) are supported by Danish Cancer Society (Denmark); German Cancer Aid, German Cancer Research Center (DKFZ), Federal Ministry of Education and Research (BMBF), Deutsche Krebshilfe, Deutsches Krebsforschungszentrum and Federal Ministry of Education and Research (Germany); the Hellenic Health Foundation (Greece); Associazione Italiana per la Ricerca sul Cancro-AIRC-Italy and National Research Council (Italy); Dutch Ministry of Public Health, Welfare and Sports (VWS), Netherlands Cancer Registry (NKR), LK Research Funds, Dutch Prevention Funds, Dutch ZON (Zorg Onderzoek Nederland), World Cancer Research Fund (WCRF), Statistics Netherlands (The Netherlands); Health Research Fund (FIS), PI13/00061 to Granada; , PI13/01162 to EPIC-Murcia), Regional Governments of Andalucía, Asturias, Basque Country, Murcia and Navarra, ISCIII RETIC (RD06/0020) (Spain); Swedish Cancer Society, Swedish Research Council and County Councils of Skåne and Västerbotten (Sweden); Cancer Research UK (14136 to EPIC-Norfolk; C570/A16491 and C8221/A19170 to EPIC-Oxford), Medical Research Council (1000143 to EPIC-Norfolk, MR/M012190/1 to EPIC-Oxford) (United Kingdom). For information on how to submit an application for gaining access to EPIC data and/or biospecimens, please follow the instructions at http://epic.iarc.fr/access/index.php

ESTHER
The ESTHER study was supported by a grant from the Baden Württemberg Ministry of Science, Research and Arts. The ESTHER group would like to thank Hartwig Ziegler, Sonja Wolf, Volker

Hermann, Heiko Müller, Karina Dieffenbach, Katja Butterbach for their valuable contributions to the study.

FHCRC
The FHCRC studies were supported by grants R01-CA056678, R01-CA082664, and R01-CA092579 from the US National Cancer Institute, National Institutes of Health, with additional support from the Fred Hutchinson Cancer Research Center. We thank all the men who participated in these studies.

IPO-Porto
The IPO-Porto study was funded by Fundação para a Ciência e a Tecnologia (FCT; UIDP/0076/2020, CEECINST/00091/2018, and 2021.03835.CEECIND) and by IPO-Porto Research Center (CI-IPOP-24-2015). We would like to express our gratitude to all patients and families who have participated in this study.

MAYO
The Mayo group was supported by the US National Cancer Institute (R01CA72818)

MCCS
The Melbourne Collaborative Cohort Study (MCCS) cohort recruitment was funded by VicHealth and Cancer Council Victoria. The MCCS was further supported by Australian National Health and Medical Research Council grants 209057 and 396414 and by infrastructure provided by Cancer Council Victoria. Cases and their vital status were ascertained through the Victorian Cancer Registry and the Australian Institute of Health and Welfare, including the National Death Index and the Australian Cancer Database. The Prostate Cancer Program of Cancer Council Victoria also acknowledge grant support from The National Health and Medical Research Council, Australia (126402, 209057, 251533, 396414, 450104, 504700, 504702, 504715, 623204, 940394, 614296,), VicHealth, Cancer Council Victoria, The Prostate Cancer Foundation of Australia, The Whitten Foundation, PricewaterhouseCoopers, and Tattersall's.

MEC
The MEC was supported by NIH grants CA63464, CA54281, CA098758, and CA164973.

MOFFITT
The Moffitt group was supported by the US National Cancer Institute (R01CA128813, PI: J.Y. Park).

PCMUS
The PCMUS study was supported by the Bulgarian National Science Fund, Ministry of Education and Science (contract DOO-119/2009; DUNK01/2-2009; DFNI-B01/28/2012) with additional support from the Science Fund of Medical University - Sofia (contract 51/2009; 8I/2009; 28/2010; ).

ProtecT
ProtecT would like to acknowledge the support of The University of Cambridge, Cancer Research UK. Cancer Research UK grants [C8197/A10123] and [C8197/A10865] supported the genotyping team. We would also like to acknowledge the support of the National Institute for Health Research, which funds the Cambridge Bio-medical Research Centre, Cambridge, UK. We would also like to acknowledge the support of the National Cancer Research Prostate Cancer: Mechanisms of Progression and Treatment (PROMPT) collaborative (grant code G0500966/75466), which has funded tissue and urine collections in Cambridge. We are grateful to the staff at the Welcome Trust Clinical Research Facility, Addenbrooke's Clinical Research Centre, Cambridge, UK, for their help in conducting the ProtecT study. We also acknowledge the support of the NIHR Cambridge Biomedical Research Centre, the DOH HTA (ProtecT grant), and the NCRI / MRC (ProMPT grant) for help with the bio-repository. The UK Department of Health funded the ProtecT study through the NIHR Health Technology

Assessment Programme (projects 96/20/06, 96/20/99). The ProtecT trial and its linked ProMPT and CAP (Comparison Arm for ProtecT) studies are supported by the Department of Health, England; Cancer Research UK grant number C522/A8649, Medical Research Council of England grant number G0500966, ID 75466 and The NCRI, UK. The epidemiological data for ProtecT were generated through funding from the Southwest National Health Service Research and Development. DNA extraction in ProtecT was supported by USA Dept of Defense award W81XWH-04-1-0280, Yorkshire Cancer Research, and Cancer Research UK. The authors would like to acknowledge the contribution of all members of the ProtecT study research group. The views and opinions expressed therein are those of the authors and do not necessarily reflect those of the Department of Health of England. The bio-repository from ProtecT is supported by the NCRI (ProMPT) Prostate Cancer Collaborative and the Cambridge BMRC grant from NIHR. We acknowledge support from the National Cancer Research Institute (National Institute of Health Research (NIHR) Collaborative Study: "Prostate Cancer: Mechanisms of Progression and Treatment (PROMPT)" (grant G0500966/75466). We thank the National Institute for Health Research, Hutchison Whampoa Limited, the Human Research Tissue Bank (Addenbrooke's Hospital), and Cancer Research UK. The authors would like to thank those men with prostate cancer and the subjects who have donated their time and their samples to the Cambridge Biorepository, which was used in this research. We also would like to acknowledge the support of the research staff in S4, who so carefully curated the samples and the follow-up data (Jo Burge, Marie Corcoran, Anne George, and Sara Stearn).

QLD
The QLD research is supported by The National Health and Medical Research Council (NHMRC) Australia Project Grants [390130, 1009458] and NHMRC Career Development Fellowship, Cancer Australia PdCCRS, and Cancer Council Queensland funding to J Batra. The QLD team would like to acknowledge and sincerely thank the urologists, pathologists, data managers, and patient participants who have generously and altruistically supported the QLD cohort.

SEARCH
SEARCH is funded by a programme grant from Cancer Research UK [C490/A10124] and supported by the UK National Institute for Health Research Biomedical Research Centre at the University of Cambridge. The University of Cambridge has received salary support in respect of PP from the NHS in the East of England through the Clinical Academic Reserve.

TAMPERE
The Tampere (Finland) study was supported by the Academy of Finland (251074), The Finnish Cancer Organisations, the Sigrid Juselius Foundation, and the Competitive Research Funding of the Tampere University Hospital (X51003). The PSA screening samples were collected by the Finnish part of ERSPC (European Study of Screening for Prostate Cancer). TAMPERE would like to thank Riina Liikanen, Liisa Maeaettaenen, and Kirsi Talala for their work on samples and databases.

UKGPCS
UKGPCS would also like to thank the following for funding support: The Institute of Cancer Research and The Everyman Campaign, The Prostate Cancer Research Foundation, Prostate Research Campaign UK (now Prostate Action), The Orchid Cancer Appeal, The National Cancer Research Network UK, The National Cancer Research Institute (NCRI) UK. We are grateful for support of NIHR funding to the NIHR Biomedical Research Centre at The Institute of Cancer Research and The Royal Marsden NHS Foundation Trust. UKGPCS should also like to acknowledge the NCRN nurses, data managers and Consultants for their work in the UKGPCS study. UKGPCS would like to thank all urologists and other persons involved in the planning, coordination, and data collection of the study. KM and AL were in part supported by the NIHR Manchester Biomedical Research Centre.

ULM
The Ulm group received funds from the German Cancer Aid (Deutsche Krebshilfe).

UTAH
The Keith and Susan Warshaw Fund, C. S. Watkins Urologic Cancer Fund, and The Tennity Family Fund supported the Utah study. The project was supported by Award Number P30CA042014 from the National Cancer Institute.

WUGS
WUGS would like to thank the following for funding support: The Anthony DeNovi Fund, the Donald C. McGraw Foundation, and the St. Louis Men's Group Against Cancer.
